# Direct Evidence for Microdomain-Specific Localization and Remodeling of Functional L-Type Calcium Channels in Rat and Human Atrial Myocytes

**DOI:** 10.1161/CIRCULATIONAHA.115.018131

**Published:** 2015-12-21

**Authors:** Alexey V. Glukhov, Marina Balycheva, Jose L. Sanchez-Alonso, Zeki Ilkan, Anita Alvarez-Laviada, Navneet Bhogal, Ivan Diakonov, Sophie Schobesberger, Markus B. Sikkel, Anamika Bhargava, Giuseppe Faggian, Prakash P. Punjabi, Steven R. Houser, Julia Gorelik

**Affiliations:** From Department of Cardiovascular Sciences, National Heart and Lung Institute, Imperial College London, United Kingdom (A.V.G., M.B., J.L.S.-A., Z.I., A.A.-L., N.B., I.D., S.S., M.B.S., A.B., P.P.P., J.G.); University of Verona, School of Medicine, Verona, Italy (M.B., G.F.); Department of Cardiothoracic Surgery, Hammersmith Hospital, National Heart and Lung Institute, Imperial College London, United Kingdom (P.P.P.); and Cardiovascular Research Center and Department of Physiology, Temple University School of Medicine, Philadelphia, PA (S.R.H.).

**Keywords:** calcium channels, heart atria, heart failure, myocytes, cardiac, scanning ion conductance microscopy, T-tubules

## Abstract

Distinct subpopulations of L-type calcium channels (LTCCs) with different functional properties exist in cardiomyocytes. Disruption of cellular structure may affect LTCC in a microdomain-specific manner and contribute to the pathophysiology of cardiac diseases, especially in cells lacking organized transverse tubules (T-tubules) such as atrial myocytes (AMs).

**Methods and Results—:**

Isolated rat and human AMs were characterized by scanning ion conductance, confocal, and electron microscopy. Half of AMs possessed T-tubules and structured topography, proportional to cell width. A bigger proportion of myocytes in the left atrium had organized T-tubules and topography than in the right atrium. Super-resolution scanning patch clamp showed that LTCCs distribute equally in T-tubules and crest areas of the sarcolemma, whereas, in ventricular myocytes, LTCCs primarily cluster in T-tubules. Rat, but not human, T-tubule LTCCs had open probability similar to crest LTCCs, but exhibited ≈40% greater current. Optical mapping of Ca^2+^ transients revealed that rat AMs presented ≈3-fold as many spontaneous Ca^2+^ release events as ventricular myocytes. Occurrence of crest LTCCs and spontaneous Ca^2+^ transients were eliminated by either a caveolae-targeted LTCC antagonist or disrupting caveolae with methyl-β-cyclodextrin, with an associated ≈30% whole-cell *I*_Ca,L_ reduction. Heart failure (16 weeks post–myocardial infarction) in rats resulted in a T-tubule degradation (by ≈40%) and significant elevation of spontaneous Ca^2+^ release events. Although heart failure did not affect LTCC occurrence, it led to ≈25% decrease in T-tubule LTCC amplitude.

**Conclusions—:**

We provide the first direct evidence for the existence of 2 distinct subpopulations of functional LTCCs in rat and human AMs, with their biophysical properties modulated in heart failure in a microdomain-specific manner.

## Abstract 2

Supplemental Digital Content is available in the text.

In the heart, L-type calcium channels (LTCCs) are essential in determining the electric and mechanical properties of cardiac muscle.^1^ In adult ventricular myocytes, LTCCs are predominantly located in the transverse tubules (T-tubules),^2^ where they form dyadic complexes with calcium-sensing and -release units, the ryanodine receptors (RyR2s) on the opposing junctional sarcoplasmic reticulum membrane (SR). A well-developed network of ventricular T-tubules ensures that the electric impulse is conducted into the cell interior, where Ca^2+^ influx can trigger the opening of RyR2 and subsequent release of SR Ca^2+^ stores. Atrial myocytes are believed to lack an elaborate T-tubule network^3–5^ and their Ca^2+^ signaling is substantially different from that in ventricular myocytes.^6–8^ Lack of a regular T-tubular system has been thought to affect the distribution of LTCCs and give rise to the unique Ca^2+^ signaling in atrial myocytes.^6,9,10^

**Clinical Perspective on p 2384**

A number of important LTCC subpopulations have been identified in ventricular myocytes that associate with unique macromolecular signaling complexes and scaffolding proteins, which enables the modulation of Ca^2+^ signaling.^11^ Although the main population of LTCCs is localized to dyadic junctions, extradyadic channels are also associated with the surface membrane.^12,13^ Caveolin-3 (Cav3)–rich signaling microdomains are found to harbor specific LTCCs that may play an important role in modulation of Ca^2+^ signaling, particularly in cells lacking organized T-tubules such as atrial^5,14–16^ and neonatal^17^ myocytes. However, until recently, it was difficult to test this hypothesis because of the lack of appropriate experimental approaches.

The spatial compartmentation of Ca^2+^-signaling complexes was first assessed by immunofluorescence microscopy,^18^ but imaging does not provide information on the functionality of channel proteins within a subcellular domain. Recent methodological advances have made it possible to image the topography of a live cardiomyocyte and study the clustering of functional ion channels within specific microdomains.^2^ Here, we used a super-resolution scanning patch-clamp technique to study the distribution of functional LTCCs in different structural microdomains of the sarcolemma of rat and human atrial myocytes. We provide direct evidence for 2 distinct subpopulations of atrial LTCCs: one localized in the T-tubules and another linked to caveolae structures. Our findings demonstrate that LTCCs, which are located in caveolae, critically contribute to atrial Ca^2+^ signaling, particularly in cardiomyocytes lacking an organized T-tubule network. These different LTCC subpopulations may underlie the regional heterogeneity of Ca^2+^ signaling and susceptibility to spontaneous Ca^2+^ release events in the atrial. Using a rat model of heart failure (HF), we highlight the relevance of the concept of microdomain-specific remodeling of LTCCs: a disruption in the delicate balance of dynamic interactions between dyadic LTCCs and their cellular microenvironment can alter Ca^2+^ signaling and lead to pathological changes in cellular physiology.

## Materials and Methods

Detailed methods are provided in the online-only Data Supplement Methods.

All studies complied with the United Kingdom Home Office regulation governing the care and use of laboratory animals and with the *Guide for the Care and Use of Laboratory Animals* published by the US National Institutes of Health (NIH publication No. 85-23, revised 1996).

### Myocyte Isolation, T-Tubule Characterization, and Animal Models

Single atrial myocytes were isolated separately from both left (LA) and right (RA) atrial of control and 16-week post–myocardial infarction rats (online-only Data Supplement Table I). The subcellular T-tubule system was visualized by confocal imaging of Di-8-ANEPPS–stained cells. Surface topography was characterized by scanning ion conductance microscopy which uses a glass nanopipette as a sensitive probe.^19^

### Super-resolution Scanning Patch-Clamp With Pipette Clipping Modification

After generating a topographical image of the cell surface by scanning ion conductance microscopy, the tip diameter of the pipette was widened by clipping^19^ to increase the area of attachment. The pipette was then lowered to a specific location until it touched the membrane, and a high-resistance seal was established. Recordings were then performed in a cell-attached mode. Controlled widening of the scanning nanopipette tip is described in detail in the online-only Data Supplement Methods. Macroscopic calcium currents were recorded by using the whole-cell patch-clamp technique.^20^

### Optical Mapping and Data Analysis

Optical mapping of cells loaded with the Ca^2+^-sensitive fluorescent dye Fluo-4 AM via a complementary metal-oxide semiconductor camera ULTIMA-L (SciMedia, USA Ltd, Costa Mesa, CA) was used to monitor localized changes in [Ca^2+^]_*i*_.

### Statistical Analysis

All graphs and statistical analysis were performed by using either GraphPad prism 5 or Origin version 6.1. The average values were calculated throughout all cells studied within the groups and then compared between the groups. Normality was tested using the Kolmogorov-Smirnov test. In cases where data failed the normality test, the nonparametric Mann-Whitney test was used instead of the unpaired Student *t* test, and the nonparametric Kruskal-Wallis test was used instead of analysis of variance. Statistical differences were assessed with analysis of variance, Student *t* test, Mann-Whitney test, Kruskal-Wallis test, χ^2^, and Fisher exact test as appropriate. All data are expressed as mean±standard error of the mean. A value of *P*<0.05 was considered statistically significant.

## Results

### In Situ T-Tubule Imaging in Isolated Rat Atrial Preparations

**Figure 1.**
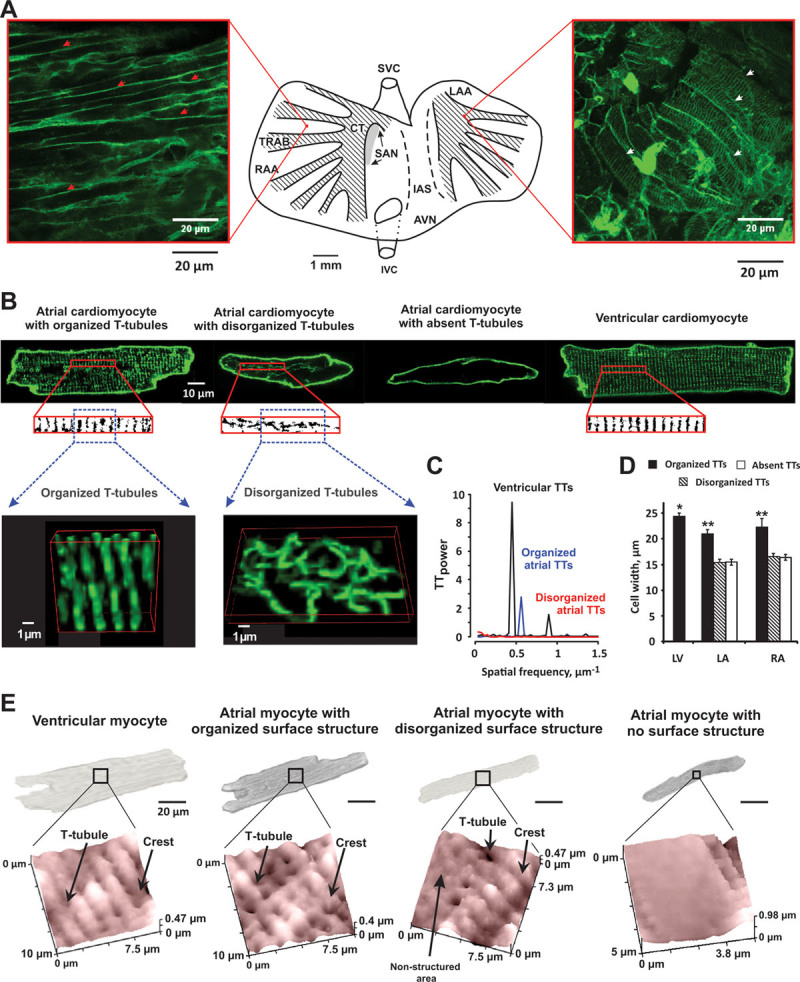
Spatial heterogeneity of the atrial T-tubular system: in situ and in vitro measurements. **A**, In situ confocal imaging of T-tubules (TTs) in intact rat atrial preparation stained with wheat germ agglutinin. In the middle, the schematic outlines of the isolated rat atrial preparation showing the main anatomic features. The enlarged images from the endocardium of the right (RAA) and left (LAA) atrial appendages demonstrate typical atrial myocytes with organized TTs (white arrows), disorganized TTs (red arrows), or mixture of both types. AVN indicates atrioventricular node; CT, crista terminalis; IAS, interatrial septum; IVC, inferior vena cava; SAN, sinoatrial node; SVC, superior vena cava; and TRAB, trabeculae. **B**, Di-8-ANEPPS membrane staining showing a TT network in ventricular myocytes and in atrial myocytes with organized, disorganized, and absent TTs. Below the confocal images, enlarged areas of 40×5 µm are shown that were binarized and used in TT density and regularity measurements. For structured atrial myocytes, 3D reconstructions of the TTs obtained from confocal stack images are shown (see also online-only Data Supplement Movies I and II). **C**, Representative distribution of power of the predominant frequency retrieved from 2D Fourier transformation of confocal images (**B** insets). **D**, Average width of cells with different TT structure isolated from left ventricle (LV, n=45 cells from 3 rats) and LA and RA (n=29/2, 26/35, and 22/38 cells from 6 rats for organized, disorganized, and absent TTs for cells from LA/RA, respectively). * P<0.001 versus atrial myocytes; ** *P*<0.001 versus other cell groups within the atrium by unpaired Student *t* test. **E**, Correlation between surface structure and cell size. Optical images and topography scans (zoomed areas) of a ventricular myocyte and atrial myocytes with various degrees of organization of surface structures are shown. TTs, crests, and nonstructured areas are indicated by arrows. Note that the cell shown in the right-most panel does not possess any organized surface structures.

To characterize the atrial T-tubular network, we performed in situ T-tubule imaging on intact rat atrial using whole-mount fluorescence labeling with glycophilic lectin wheat germ agglutinin. We found significant region-dependent heterogeneity in T-tubule structure throughout the atrial. Although the LA myocardium predominantly consisted of cardiomyocytes with T-tubules (Figure 1A, Right), in the RA, we observed 3 groups of cardiomyocytes: cells (1) with organized T-tubules, (2) with disorganized T-tubules, and (3) with absent T-tubules (Figure 1A, Left).

### Subcellular T-Tubule System in Rat Atrial Cardiomyocytes

Confocal imaging of Di-8-ANEPPS–stained cardiomyocytes isolated separately from the LA and RA revealed that, although about one-third of cells do not have T-tubules (≈39% in RA; 34% in LA), other cardiomyocytes possess a T-tubular network of some sort. We also found cells with organized T-tubular networks similar to those found in ventricular myocytes, and those with disorganized T-tubules (Figure 1B). The majority of atrial myocytes with organized T-tubules were isolated from the LA (≈40% versus 2% in LA versus RA). Conversely myocytes isolated from the RA were more likely to have disorganized T-tubule network (26% versus 59% in LA versus RA) (online-only Data Supplement Figure I). In ventricular rat myocytes, T-tubules are distributed regularly at ≈2-µm intervals, as demonstrated by Fourier transform of binarized images of T-tubules (Figure 1C). In contrast, the atrial T-tubular network is less dense and less regular than in ventricular myocytes. Atrial myocytes with organized T-tubules showed a smaller, but still distinct peak in the Fourier transform plot (3.1±0.6 AU versus 11.3±0.9 AU in atrial (n=31 cells from 4 rats) versus ventricular myocytes (n=45 cells from 3 rats), *P*<0.001). On the other hand, atrial cells with disorganized or absent T-tubules (both in LA and RA) did not show a dominant peak on the Fourier transform plot (Figure 1C). Organization of the atrial T-tubular network correlates with cell width: cells showing organized T-tubular networks were larger than cells with disorganized or absent T-tubules (Figure 1D and online-only Data Supplement Figure II).

### Surface Structures in Rat Atrial Myocytes

Our previous scanning ion conductance microscopy imaging of rat ventricular myocytes^21^ has clearly showed the surface topography to be structured with T-tubule openings arranged along Z-grooves and the domed crests located in between them. To characterize cardiomyocyte topography, we have introduced the Z-groove index^21^: a ratio of the observed Z-groove length to the total extrapolated Z-groove length (as if they were present throughout the entire surface). We applied this analysis to characterize topography of atrial myocytes. We found that the larger an atrial myocyte, the more regular its surface topography. This is consistent with the more organized T-tubule network noted in these larger myocytes. Atrial myocytes with organized surface structures and apparent T-tubule openings, similar to those seen in ventricular myocytes, were wider than those with patchy nonstructured areas on their surface, which in turn were wider than atrial myocytes that entirely lacked surface structures (Figure 1E). Similarly, the Z-groove index was significantly higher in LA versus RA myocytes (0.66±0.03 versus 0.50±0.03 in LA versus RA, respectively, *P*<0.001), confirming the presence of more structured myocytes in the LA.

### Rat Atrial Myocyte Ca^2+^ Signaling

**Figure 2.**
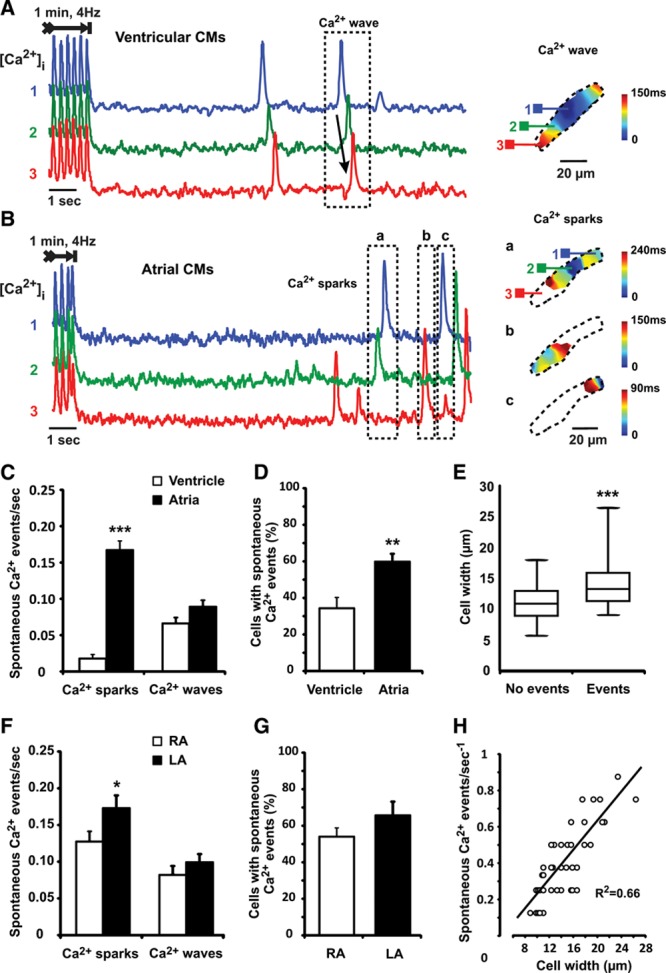
Spontaneous Ca^2+^ release events. Spontaneous Ca^2+^ activity was measured in isolated ventricular (**A**) and atrial (**B**) myocytes. Cells were electrically paced at 4 Hz for 1 minute to enhance sarcoplasmic reticulum Ca^2+^ loading. Ca^2+^ sparks and waves were quantified during a 8- to 16-s rest period after cessation of pacing. On the left, optical traces indicating changes in [Ca^2+^]_*i*_ during measurements are shown from 3 different areas (1–3) from the selected cardiomyocytes. On the right, Ca^2+^ transient propagation color contour maps are presented for a spontaneous Ca^2+^ wave recorded from the ventricular myocyte (**A**) and 3 Ca^2+^ sparks obtained from the atrial myocyte (**B**). Near the maps, the corresponding color time scales for propagation time are shown. **C**, Average frequency of spontaneous Ca^2+^ sparks and waves measured in ventricular (n=126 from 8 rats) and atrial (n=357 from 9 rats) myocytes. *** *P*<0.001 by Mann-Whitney test. **D**, Percentage of atrial (n=126) and ventricular myocytes (n=357) with spontaneous Ca^2+^ release events. ** *P*<0.01 by unpaired Student *t* test. **E**, Average cell width for atrial myocytes with and without spontaneous Ca^2+^ release events (n=117 cells from 7 rats). *** *P*<0.001 by Mann-Whitney test. **F**, Average frequency of spontaneous Ca^2+^ sparks and waves measured separately in right (RA, n=156 from 4 rats) and left (LA, n=201 from 4 rats) atrial myocytes. * *P*<0.05 by Mann-Whitney test. **G**, Percentage of RA (n=156) and LA myocytes (n=201) with spontaneous Ca^2+^ events. **H**, Correlation between cell width and frequency of spontaneous Ca^2+^ events for atrial myocytes together with a correlation coefficient. CM indicates cardiomyocyte; LA, left atrium; and RA, right atrium.

Atrial myocytes differ strikingly from ventricular myocytes in shape, magnitude, and kinetics of subcellular Ca^2+^ transients and in the dynamic of spontaneous Ca^2+^ release events, as well.^4,6^ These differences were hypothesized to be largely attributed to the distinct structure of atrial T-tubules and to altered distribution of LTCCs and their coupling to RyR, as well.^10^ Optical mapping of spontaneous Ca^2+^ activity revealed that atrial myocytes have different Ca^2+^ cycling than ventricular myocytes. Among all spontaneous Ca^2+^ release events, Ca^2+^ waves propagating throughout the entire cell (Figure 2A) were distinguished from nonpropagating Ca^2+^ release events (Figure 2B). Although we did not distinguish Ca^2+^ sparks from Ca^2+^ puffs,^22,23^ we observed 2 groups of nonpropagating Ca^2+^ events: one with a smaller amplitude (23±1% of the amplitude of the corresponding paced Ca^2+^ transients, 60% of all nonpropagating Ca^2+^ events) and another with a higher amplitude (60±4% of the paced Ca^2+^ transient amplitude, 40% of all nonpropagating Ca^2+^ events), which could be attributed to Ca^2+^ puffs and Ca^2+^ sparks, respectively.^22^ In comparison with ventricular myocytes, atrial cells showed a significantly higher frequency of nonpropagating Ca^2+^ release events following a period of high-frequency stimulation (Figures 2C and 2D and online-only Data Supplement Figure III).

We found that wider rat atrial myocytes exhibited a significantly higher number of spontaneous Ca^2+^ release events (Figure 2E). No spontaneous Ca^2+^ release events were observed in cells thinner than 11.0±0.4 µm (*P*=0.002 versus cells with events, Figure 2E). In addition, nonpropagating Ca^2+^ release events were observed in atrial myocytes 13.2±0.6 µm wide, whereas propagating Ca^2+^ waves were found in cells ≥14.9±1.1 µm wide (data not shown). Cardiomyocytes isolated from the LA demonstrated a higher number of Ca^2+^ release events than RA myocytes (Figures 2F and 2G). In atrial myocytes that exhibited spontaneous Ca^2+^ release events, the number of events was found to be proportional to cell width, as shown in Figure 2H.

### Spatial Localization of Functional LTCCs

**Figure 3.**
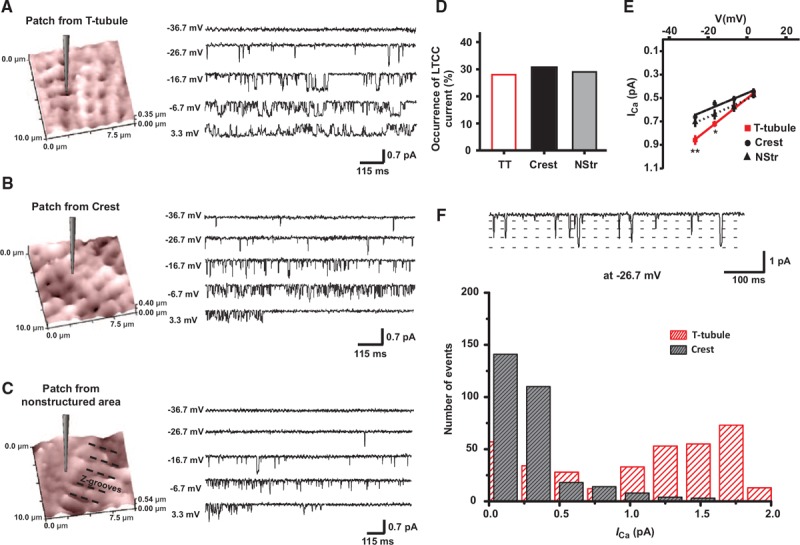
Single LTCC activity recorded from T-tubule, crest, and nonstructured areas in rat atrial myocytes. Typical 10×10 µm topographical scans of cardiomyocytes showing locations where a pipette was placed after clipping and a giga-seal was obtained over a T-tubule (**A**), a crest (**B**), and a nonstructured flat area (**C**) of the sarcolemma. On the right, corresponding representative current traces of single LTCC activity at the given voltages using a pipette of 25 Ω resistance. **D**, Percentage of occurrence of the LTCC current from the T-tubules (TT; 78 successful patches), crests (63 successful patches), and nonstructured areas (NStr; 26 successful patches). **E**, Current-voltage relationship of single LTCC activity recorded from the T-tubules, crest, and NStr areas . n=6 to 16 channels for T-tubules, n=8 to 12 channels for crests, and n=5 channels for nonstructured areas. * *P*<0.05 and ** *P*<0.01 versus C-LTCCs by analysis of variance. **F, Top**, LTCC openings evoked by voltage jumps to –26.7mV and using 90 mM Ba^2+^ as the charge carrier. The dashed lines indicate substates and fully open levels. **Bottom**, The LTCC amplitude histogram of single-channel openings to different substate levels measured as shown on the panel above. C-LTCC indicates crest L-type calcium channel; and LTCC, L-type calcium channel.

In adult rat ventricular myocytes, functional LTCCs are predominantly localized in the T-tubules.^2^ Here, we applied the same super-resolution scanning patch-clamp method^2^ (Figures 3A through 3C) and found that, in contrast to ventricular myocytes, in rat atrial cells LTCCs were recorded with similar frequency from T-tubules (T-LTCC), crests (C-LTCC), and nonstructured areas (Figure 3D).

Although no difference in LTCC open probability was found in different locations (*P*[open] at –6.7mV: 0.060±0.006 for T-LTCCs versus 0.067±0.013 for C-LTCCs, not significant [NS]; and versus 0.053±0.006 in nonstructured area, NS), T-LTCCs possessed ≈40% higher amplitude at negative voltages in comparison with the other 2 groups of channels (Figure 3E). Like many other types of channels, LTCCs show multiple subconductance levels in addition to the largest and the main open state of the channel.^24,25^ Examples of these substates are given in Figure 3F, Top, and online-only Data Supplement Figure IV. As summarized in Figure 3F, Bottom, C-LTCCs exhibited low-amplitude subconductance states more frequently than T-LTCCs: open probability of low-amplitude subconductance states was 0.027±0.005 and 0.005±0.002 for C-LTCCs (n=4) and T-LTCCs (n=5), respectively, *P*<0.01.

**Figure 4.**
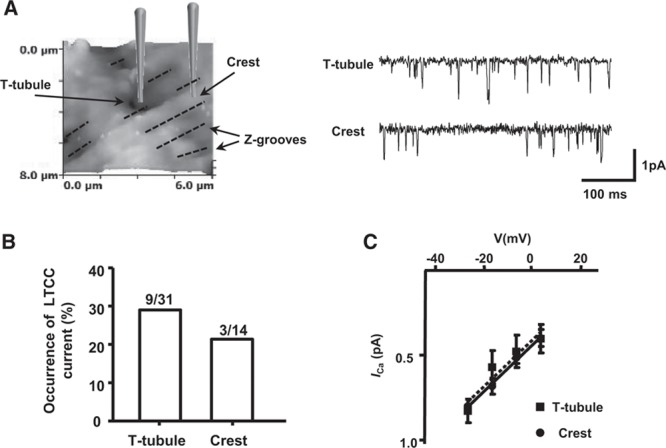
Microdomain distribution of functional LTCCs in human right atrial myocytes. **A, Left**, Typical 10×10 µm topographical scan of the human right atrial myocyte isolated from a patient with sinus rhythm. T-tubule and crest areas are indicated by arrows. Z-grooves are shown by the dotted lines when present. **Right**, Representative current traces of single LTCC activity recorded at –6.7mV using a pipette of 25 Ω resistance. **B**, Percentage of occurrence of functional LTCC current from the T-tubules and crest areas of human atrial myocytes. Above the columns is the number of patches with the active current out of the total number of patches for each location. **C**, Current-voltage relationship of single LTCC current activity recorded from the T-tubules and from the crests. LTCC indicates L-type calcium channel.

In addition, we explored microdomain-specific distribution of functional LTCCs in several human RA samples (Figure 4A). On average, human atrial myocytes had a Z-groove index of 0.57±0.02 (n=27), which is similar to that measured in rat RA. Similar to rat, human LTCCs were recorded with similar frequency from T-tubules and crest (Figure 4B). We did not observe any difference in the voltage-current characteristics between the areas (Figure 4C). However, in contrast to rat, human T-LTCCs had significantly higher open probability than C-LTCCs: *P*(open) at –6.7mV: 0.03±0.002 for T-LTCCs versus 0.017±0.001 for C-LTCCs, *P*<0.001.

### Caveolae as a Source of Extradyadic LTCCs

It has been demonstrated in ventricular myocytes that some LTCCs could be housed in caveolae.^12,13^ We hypothesized that atrial extratubular LTCCs recorded from the crests and nonstructured areas might be localized to caveolae. To address this hypothesis, we used 2 different approaches to disrupt caveolae: treatment with methyl-β-cyclodextrin (MβCD) and direct LTCC inhibition in Cav3-containig membranes using a specific peptide inhibitor Rem.

**Figure 5.**
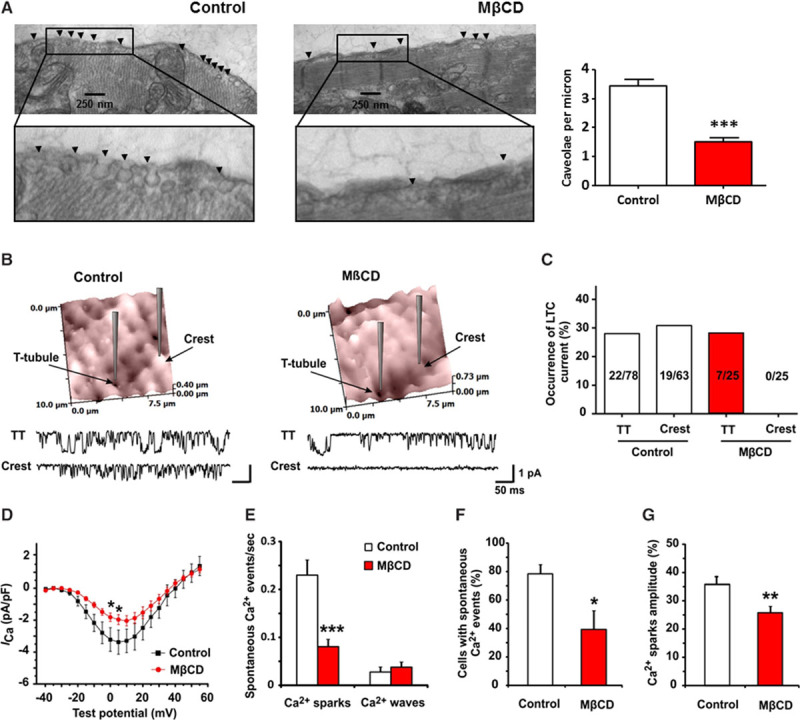
Cholesterol depletion removes caveolae, abolishes the occurrence of extratubular LTCCs decreasing whole-cell *I*_Ca,L_ and suppressing spontaneous Ca^2+^ sparks. **A**, Ultrastructural changes in rat atrial myocytes after methyl-β-cyclodextrin incubation. Electron micrographs of representative control (**Left**) and 10 mM methyl-β-cyclodextrin (MβCD)–treated rat atrial (**Right**) myocytes are shown. Caveolae are marked by arrowheads. **Right**, Caveolae per micrometer in the cellular membrane before and after MβCD treatment (n=16 for control and n=10 for MβCD group, n=3 rats per group). *** *P*<0.001 by unpaired Student *t* test. **B**, Typical 10×10 µm topographical scans of control (**Left**) and MβCD-treated (**Right**) rat atrial myocytes. Below are single-channel recordings obtained from the T-tubule (TT) and the crest of sarcolemma (Crest). **C**, Percentage of LTCC current occurrence in the T-tubules and crests. ** *P*<0.01 for Crest MβCD LTCC versus other groups, by Fisher exact test. **D**, Whole-cell *I*_Ca,L_ density (n=9 for control and n=12 for MβCD groups) before and after MβCD treatment. * *P*<0.05 by unpaired Student *t* test. **E**, Along with changes in *I*_Ca,L_, MβCD significantly suppressed the occurrence of spontaneous Ca^2+^ sparks. *** *P*<0.001 by Mann-Whitney test. The proportion of cells with spontaneous Ca^2+^ events (**F**) and Ca^2+^ spark amplitude (**G**) were also decreased following the treatment. Ca^2+^ spark amplitude was calculated as a percentage from the amplitude of electrically induced Ca^2+^ transient measured during 4-Hz pacing. n=82 cells from 12 rats and n=99 cells from 7 rats for control and MβCD groups, respectively. * *P*<0.05, ** *P*<0.01 by unpaired Student *t* test. LTCC indicates L-type calcium channel.

Incubation of atrial myocytes with 10 mM MβCD for 30 minutes at room temperature^17,26^ resulted in ≈60% depletion of caveolae (Figure 5A), with no changes in cell topography and T-tubule density (online-only Data Supplement Figure V). While MβCD did not affect LTCCs occurrence in the T-tubules (28% versus 28%, before and after MβCD, NS), it completely abolished the occurrence of extratubular LTCCs (30.1% versus 0% before and after MβCD, respectively) (Figures 5B and 5C), suggesting a crucial role of caveolae for spatial compartmentation of LTCCs in atrial myocytes. No changes in either the open probability or the current-voltage relationship of T-LTCCs were observed after MβCD (online-only Data Supplement Figure VI). Associated with LTCC removal from the crest, MβCD significantly decreased the whole-cell calcium current (*I*_Ca,L_) density by ≈30% (Figure 5D) without affecting cell capacitance (73.9±14.6 pF for control [n=9] versus 72.6±5.8 pF for MβCD [n=13] groups, NS). In contrast, in ventricular myocytes, no changes in either cell capacitance (136.9±8.1 pF for control versus 112.1±9.5 pF for MβCD groups, n=10/group, *P*=0.062) or *I*_Ca,L_ was observed after MβCD treatment (online-only Data Supplement Figure VII).

### Ignition of Ca^2+^ Sparks Through Caveolae

Along with changes in LTCC distribution, MβCD significantly suppressed the occurrence of spontaneous Ca^2+^ release events in rat atrial myocytes (online-only Data Supplement Figure VIII). MβCD treatment significantly decreased the number of spontaneous Ca^2+^ sparks, but not waves (Figure 5E) and reduced the number of cells featuring spontaneous Ca^2+^ release events (Figure 5F). Moreover, removing caveolae via MβCD treatment also reduced the amplitude of Ca^2+^ sparks in atrial myocytes (Figure 5G), as has been previously demonstrated in neonatal ventricular myocytes and arterial smooth muscle cells.^17^

### Caveolae-Targeted LTCC Antagonist Eliminates Occurrence of Extradyadic LTCC Current

To confirm that extratubular LTCCs are localized to Cav3-associated caveolae structures, rather than lipid rafts, we used a Cav3-targeted LTCC-blocking agent, Rem peptide.^12^ The caveolae-targeted LTCC blocker (Rem^1-265^-Cav) was generated by molecular modification of Rem, a member of the RGK GTPase family that is known to potently inhibit LTCCs.^27^ Makarewich et al^12^ have demonstrated that in rat ventricular myocytes Rem^1-265^-Cav localizes to plasma membrane specifically within caveolin-containing lipid rafts, rather than lipid rafts in general, and does not displace molecules normally found in caveolae.

**Figure 6.**
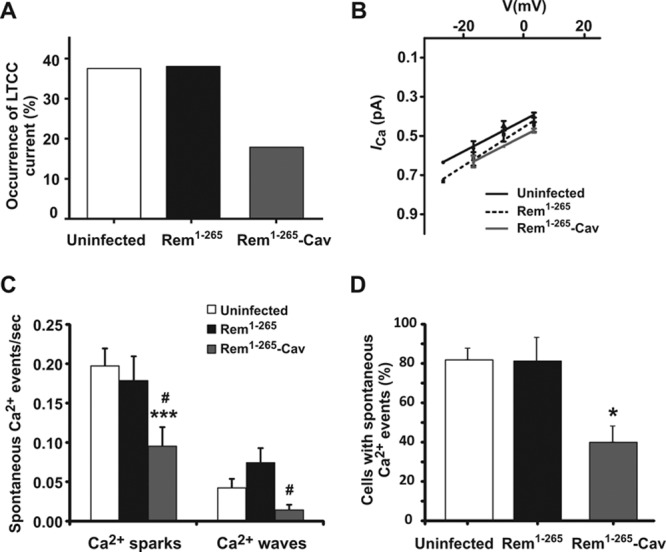
A caveolae-targeted LTCC antagonist decreases the occurrence of single LTCC current on the crest area of the sarcolemma and reduces spontaneous Ca^2+^ events. Percentage of occurrence (**A**) and current-voltage relationship (**B**) of the single LTCC current from the crest area of uninfected (48 hours of culturing without a virus; 12 channels in 32 patches), Rem^1-265^-infected (8 channels in 21 patches), and Rem^1-265^-Cav-infected (48 hours after infection; 5 channels in 28 patches) atrial myocytes. **C**, Average data for Ca^2+^ sparks and waves measured in uninfected (n=67 cells from 5 rats), Rem^1-265^-infected (n=50 cells from 4 rats), and Rem^1-265^-Cav–infected (n=35 cells from 5 rats) atrial myocytes measured 48 hours after infection. *** *P*<0.001 versus uninfected, # *P*<0.05 versus Rem^1-256^ by Kruskal-Wallis test. **D**, Percentage of atrial myocytes with spontaneous Ca^2+^ events in 3 groups of cells studied. * *P*<0.05 versus both groups by Kruskal-Wallis test. LTCC indicates L-type calcium channel.

With 60% to 65% infection efficiency, expressing Rem in rat atrial myocytes resulted in a significant decrease in the occurrence of the single LTCC on the crest area of sarcolemma (Figure 6A) without affecting LTCC biophysical properties (Figure 6B). Similarly, Rem^1-265^-Cav decreased spontaneous Ca^2+^ release events in infected cells (Figure 6C). Truncation of the membrane-docking domain in the Rem^1-265^-Cav peptide (Rem^1-265^) resulted in the inability of Rem to affect the occurrence of the single LTCC current on the crest and to change spontaneous Ca^2+^ events (Figure 6). No changes in the single channel open probability were observed.

### HF Model and Structural Changes in the Atrial

**Figure 7.**
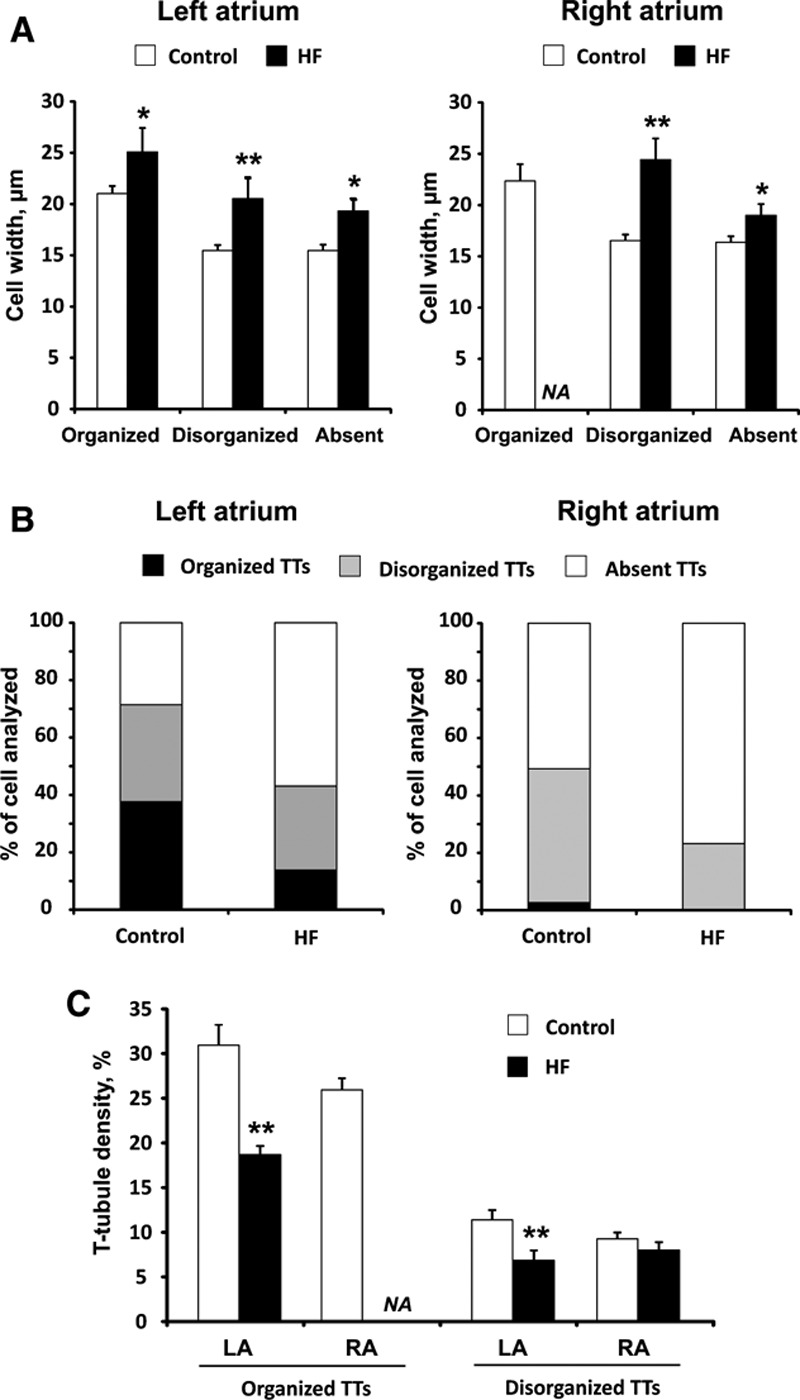
Structural remodeling of atrial myocytes in heart failure (HF) rats. Average cell width (**A**), composition (in percent) of populations of myocytes (**B**), and T-tubule system density (**C**) in cells with different T-tubule (TT) structure isolated from control and HF left (LA) and right (RA) atrial, n=77/75 control cells for LA/RA (29/26/22 and 2/35/38 cells for organized/disorganized/absent TT in LA and RA, respectively) versus n=65/43 HF cells for LA/RA (9/19/37 and 0/10/33 cells for organized/disorganized/absent TT in LA and RA, respectively) for LA/RA control versus HF rats). * *P*<0.05, ** *P*<0.01, *** *P*<0.001 versus control by unpaired Student *t* test or Mann-Whitney test as appropriate.

To study disease-associated atrial remodeling, we used the 16 weeks of post–myocardial infarction rat model of HF.^28^ This model recapitulates many features of chronic HF in patients including adverse remodeling of the organ, characterized by left ventricle dilatation, reduced ejection fraction, and raised filling pressures (online-only Data Supplement Table I). Atrial cellular hypertrophy was noted with an increase in planar width (Figure 7A).

Similar to control, in HF we also observed 3 groups of cardiomyocytes, although cells with organized T-tubules were found exclusively in the LA (Figure 7B). Despite cell hypertrophy, HF led to a profound degradation of the T-tubule system in all groups of cells, decreasing the proportion of structured myocytes and reducing the T-tubule density in both LA and RA (Figure 7C). Similarly, HF resulted in the loss of surface structures and a reduction in Z-groove index (0.57±0.01 versus 0.45±0.02 in control versus HF, *P*<0.001).

### Functional Atrial Remodeling in HF

**Figure 8.**
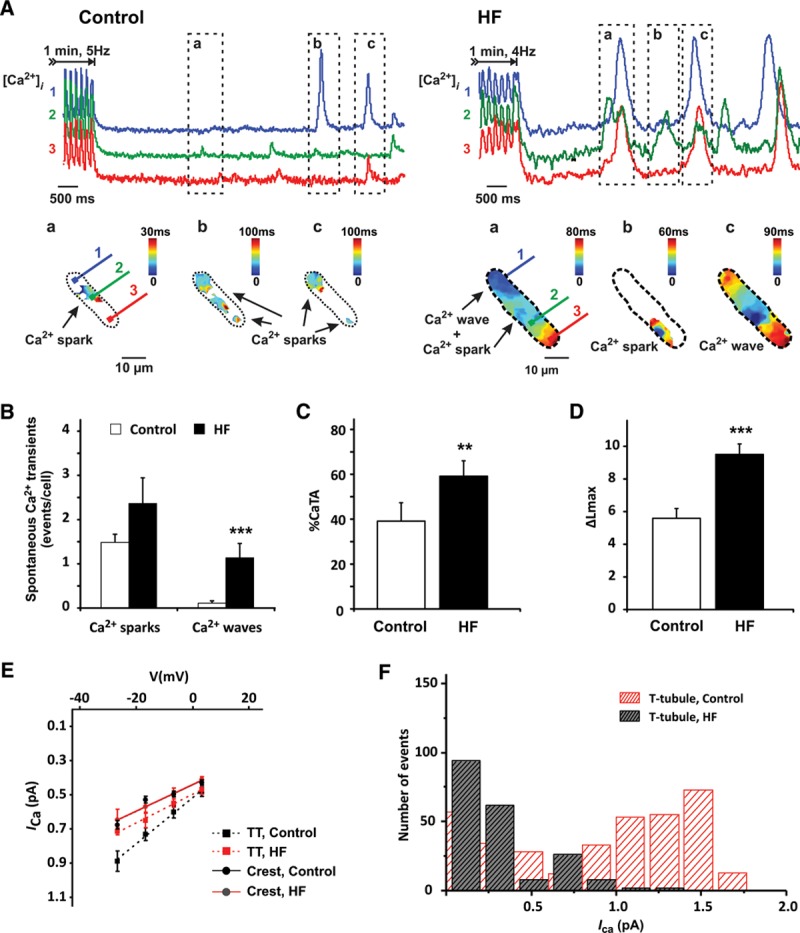
Microdomain-specific remodeling of single LTCCs in HF. **A**, Spontaneous Ca^2+^ activity measured in isolated control (**Left**) and HF (**Right**) rat atrial myocytes. Optical traces indicating changes in [Ca^2+^]_*i*_ are shown from 3 different areas (1–3) from the selected cardiomyocytes. Below the traces, Ca^2+^ transient propagation color contour maps are presented for selected events. Near the maps, the corresponding color time scales for propagation time are shown. **B**, Average frequency of spontaneous Ca^2+^ sparks and waves measured in control (n=116 cells from 5 rats) and HF (n=119 cells from 4 rats) atrial myocytes. *** *P*<0.001 by Mann-Whitney test. **C**, Maximal amplitude of sparks (in percent from a paced Ca^2+^ transient, %CaTA). ** *P*<0.01 by Mann-Whitney test. **D**, Maximal length activated by a spark (ΔL_max_, pixels). *** *P*<0.001 by unpaired Student *t* test. **E**, Current-voltage relationship of single LTCC activity recorded from the T-tubules (TT) and crest in control and HF rat atrial myocytes. **F**, LTCC amplitude histogram of single-channel openings to different substate levels calculated for T-LTCC in control and HF. CaTA indicates calcium transient amplitude; LTCC, L-type calcium channel; and T-LTCC, T-tubule L-type calcium channel.

Along with the degradation of the atrial T-tubular system, HF also caused altered Ca^2+^ cycling. We found a ≈30% increase in spontaneous Ca^2+^ spark frequency and a 3-fold increase in wave frequency (Figure 8A and 8B). The average amplitude of nonpropagated Ca^2+^ events was increased, perhaps suggesting a greater proportion of high-amplitude events (Ca^2+^ events), as opposed to low-amplitude events (Ca^2+^ puffs) (23/77 versus 60/40 for Ca^2+^ puffs/sparks in HF versus control, respectively). In addition, failing atrial myocytes were associated with slower and wider Ca^2+^ waves (Figure 8D and online-only Data Supplement Figure IX), which may result from T-tubule degradation and increase time required for dyad-to-dyad Ca^2+^ propagation.

### Microdomain-Specific Remodeling of Atrial LTCCs in HF

When characterized by the super-resolution scanning patch clamp, LTCCs in HF cells demonstrate the same equal distribution between T-tubules and crests as observed in control cells (34.3% and 30.5% versus 28% and 30.1% for T-LTCCs and C-LTCCs in HF versus control, respectively, NS). This corresponds with the similar caveolae density in HF and control myocytes (3.7±0.5 caveolae/µm in control versus 4.6±0.4 caveolae/µm in HF, *P*=0.167). Although we did not observe any significant changes in open probability for T-LTCCs and C-LTCCs in HF, we found that T-LTCCs had ≈25% smaller amplitude in HF in comparison with control T-LTCCs (Figure 8E). No changes in C-LTCC amplitude were revealed in HF. The decrease in T-LTCC amplitude in HF was associated with a change in the accessibility of the channel subconductance states (Figure 8F). In HF, the occupancy of low-amplitude substates becomes more accessible than high-amplitude substates in comparison with control (0.018±0.006 and 0.006±0.002 versus 0.006±0.002 and 0.075±0.005 for low- and high-amplitude subconductance states in HF versus control, respectively).

## Discussion

### Functional Anatomy of Atrial Myocardium

Atrial myocytes have long been perceived as having no or very few T-tubules.^3,10,29^ However, recent experimental evidence demonstrates that atrial myocytes from certain species, such as sheep,^30^ cows, horses, humans,^5^ and even rodents^4,10,15^ do possess an organized T-tubule network. In general, atrial T-tubules are sparse and less regular when compared with those in ventricular myocytes as assessed both in situ^31^ and in vitro.^10^ Our observations of T-tubules in rat atrial myocytes are consistent with those previously observed using electron microscopy^32,33^ and fluorescent membrane labeling.^4,16^ In the present study, we found significant heterogeneity of T-tubule organization between LA and RA, which might correlate with the arrangement of pectinate muscle bundles within the atrial appendages. It has been reported in rabbit atrial that myocytes isolated from the crista terminalis were significantly larger than those from the pectinate muscles, whereas the shape (the ratio of the length to the width) was found to be similar in the 2 types of cells.^34^

Anatomic heterogeneity of the T-tubular system within the atrial has been proposed to underlie heterogeneous calcium current measured within the right atrium, in addition to different expression of LTCCs. In healthy dogs, Ca^2+^ influx through LTCCs was found to be largest in crista terminalis cells, intermediate in cells from the appendage and pectinate muscles, and smallest in atrioventricular ring cells.^35^ In contrast, in rabbit right atrial myocytes isolated from different areas, whole-cell clamp recordings showed no definite variation in the density of the voltage-dependent LTCCs.^34^ Frisk and colleagues^16^ used both isolated atrial cells and tissue to demonstrate that in pig and rat atrial there was a high variability in the distribution of T-tubules and *I*_Ca,L_ among cells, with a steep dependence of *I*_Ca,L_ on atrial myocyte capacitance and T-tubule density. The authors observed more T-tubules in the epicardium than in the endocardium, which may contribute to the synchronization of contraction across the atrial wall. Thus, anatomic heterogeneity of the T-tubule network and *I*_Ca,L_ may explain complex features of atrial electric and mechanical activity including highly anisotropic physiological activation patterns,^36^ action potential duration distribution,^37^ and contractile function. In addition, disease-associated loss of T-tubules found in HF^30^ and atrial fibrillation^38^ may have an impact on calcium cycling and promotes the development of triggers of arrhythmia. One might hypothesize that distinct anatomic regions within the atrial will differentially respond on the stress and thus promote the propagation of arrhythmia triggers at specific locations aroused from superposition of areas of significant T-tubular degradation with those of profound molecular remodeling.^39^ The mechanisms that result in the development of such trigger “hot spots” require special investigation and will be the focus of our follow-up studies.

### Role of Caveolae Structures in Atrial Ca^2+^ Signaling

It has been proposed that, because of the lack of a regular T-tubule system in atrial myocytes, differential spatial distribution of LTCCs with regard to their coupling to RyR2s may underlie a unique atrial myocyte Ca^2+^-signaling process.^9,40^ In the present study, we uncovered for the first time the distinct distribution of functional atrial LTCCs in the sarcolemma where they appear at a similar frequency both in the T-tubules and the crests, in contrast to ventricular myocytes where LTCCs were primarily clustered in the T-tubules.^2^ We demonstrated the importance of the extradyadic channels, which are predominantly located in caveolae, in the regulation of Ca^2+^ signaling, particularly in cardiomyocytes lacking an organized T-tubule network. The function of LTCCs localized in caveolae remains open to question. It has been proposed that some LTCCs housed in Cav3-rich microdomains, could play an important role in the modulation of Ca^2+^ signaling. Indeed, local spontaneous Ca^2+^ release events are plentiful and, in contrast to ventricular myocytes, seem to be normally present in healthy atrial.^6,41^

Walden and colleagues demonstrated important differences in Ca^2+^-handling mechanisms between ventricular and atrial myocytes: atrial myocytes were found to have a more robust and abundant Ca^2+^ uptake mechanism and a higher SR Ca^2+^ content than ventricular myocytes.^7^ Higher SR Ca^2+^ content in atrial myocytes has been proposed to lead to Ca^2+^ overload and thus increase the sensitivity of RyR2s to cytosolic [Ca^2+^]_*i*_.^9^ This means that 1 Ca^2+^ spark is more likely to trigger another Ca^2+^ spark in atrial myocytes but remains a rare event in normal ventricular myocytes. Therefore, the benefit of the elevated SR Ca^2+^ content is that it should improve the synchrony of the atrial [Ca^2+^]_*i*_ transients when T-tubules are disorganized or absent. The downside is that in atria the elevated SR Ca^2+^ content and enhanced [Ca^2+^]_SR_ lead to the increased sensitivity of the Ca^2+^-induced Ca^2+^ release process. For thin cells, or thick cells with organized T-tubules, the Ca^2+^ signal propagation is likely to be relatively stable. However, larger myocytes with disorganized T-tubules may have an increased propensity toward subcellular Ca^2+^ alternans and thus appear to be more prone to Ca^2+^ sparks as demonstrated in our study (Figure 2). Uncontrolled [Ca^2+^]_*i*_ elevations, as occurs with Ca^2+^ alternans,^42^ will activate the Na/Ca exchanger and thereby generate Na/Ca exchanger current (*I*_NCX_) between action potentials with subsequent induction of delayed afterdepolarizations. Thus, the high SR Ca^2+^ content in atrial myocytes may be proarrhythmic. Such arrhythmogenic tendencies are unmasked in HF as cells undergo hypertrophy and the T-tubule system degrades (Figures 7 and 8). Slower and wider Ca^2+^ waves observed in failing cells in the present study would lead to prolonged depolarization time required for Na/Ca exchanger activation and thus they are more likely to result in the formation of delayed afterdepolarizations observed in failing atrial.^43,44^ Along with elevated diastolic [Ca^2+^]_*i*_ and SR Ca^2+^ overload, observed in HF, it would result in the formation of foci of triggered ectopic activity located within areas of significant T-tubular degradation.

### Unique Atrial Myocyte Ca^2+^ Signaling

It is possible that the mechanism behind the localized spontaneous Ca^2+^ release events in atrial differs from that in ventricles. In rat ventricular myocytes, 85% of all Ca^2+^ sparks evoked by electric stimulation occur within 0.5 µm of a T-tubule,^45^ and formamide-induced detubulation significantly reduces Ca^2+^ sparks in rat ventricular myocytes,^14^ suggesting an important role for T-tubules in Ca^2+^ spark initiation. Despite the broad distribution of RyRs in atrial myocytes principally along the Z-lines, most Ca^2+^ sparks occur within 1 µm of the sarcolemma.^4,8^ In contrast to ventricular myocytes, where the close (≈12 nm) proximity of RyRs and LTCCs in dyadic junctions of T-tubules facilitates Ca^2+^ release from the SR,^46^ atrial myocytes have an additional, functionally separated nonjunctional Ca^2+^ release site in the central SR not associated with T-tubules.^8,41^ Based on immunochemical experiments, these 2 kinds of atrial Ca^2+^ release sites were proposed to differ in their probability of initiating a Ca^2+^ spark, with some being designated as eager sites, whereas others normally fail to spark.^8,40^ As demonstrated by Woo et al^41^ in rat atrial myocytes, although the frequency of spontaneous unitary Ca^2+^ sparks was significantly higher in the dyads, the compound sparks, that is, localized Ca^2+^ release composed of several unitary events occurring synchronously and occupying >2 sarcomeres, appeared more prevalent in nonjunctional sites. These findings support the idea that the retarded dissipation of unitary nonjunctional focal Ca^2+^releases may facilitate the activation of neighboring release sites, leading to recruitment of a larger number of unitary that which in turn improves synchronicity of the atrial [Ca^2+^]_*i*_ transients despite their less organized T-tubule network.^9^

Nonjunctional Ca^2+^ events might be attributed to inositol-1,4,5-trisphosphate (IP_3_) dependent activation of nonjunctional RyRs.^22^ Recently, Horn et al^47^ have shown that IP_3_ can effectively modulate RyR openings and Ca^2+^ spark probability. Direct interaction between Cav3 and IP_3_-associated G_q_-protein–coupled receptor-signaling pathway has been demonstrated in canine ventricular myocytes^48^ which can link IP_3_-dependent nonjunctional Ca^2+^ events to caveolae and explain a significant decrease in occurrence of spontaneous Ca^2+^ sparks observed in atrial myocytes treated with MβCD (Figures 5E through 5G). Interestingly, a similar reduction in Ca^2+^ sparks via direct inhibition of caveolae-housed LTCCs by Rem protein (Figure 6) highlights the importance of these channels in the initiation of Ca^2+^ sparks. Alternatively, it is possible that MβCD- or Rem^1-265^-Cav–induced reduction in the whole-cell *I*_Ca,L_ decreases the steady-state SR Ca^2+^ load and thus suppresses the occurrence of spontaneous Ca^2+^ transients observed in our experiments.

### Microdomain-Specific Remodeling of Atrial LTCCs in HF

Decrease in atrial *I*_Ca,L_ in HF has been shown in both animal models^49^ and patients with congestive HF.^50^ Taking into account the T-tubule degradation, one would expect a reduction of the number of functional channels to be mainly responsible for the reduction in *I*_Ca,L_. However, as we demonstrated in the present study, an additional mechanism responsible for the reduction in *I*_Ca,L_ in failing atrial might be represented by the reduction in the amplitude of T-LTCCs. Despite the decreased *I*_Ca,L_, an increase in SR Ca^2+^ load (caffeine-induced [Ca^2+^]_*i*_ release) has been observed in failing atrial.^44,49^ In addition to the increased SR Ca^2+^ loading, a significant reduction in calsequestrin expression has been found in failing atrial,^44^ and this has been linked to increase in SR Ca^2+^ leak and atrial arrhythmogenesis, perhaps as a result of decreased SR Ca^2+^ buffering.^39^ Both SR Ca^2+^ leak and elevated diastolic [Ca^2+^]_*i*_ may affect T-LTCC current, causing a reduction in amplitude, either through Ca^2+^-dependent inactivation or phosphorylation (Figures 8E and 8F). Therefore, a disruption in the delicate balance of dynamic interactions between dyadic LTCCs and their microenvironment may alter Ca^2+^ signaling and can lead to pathological changes in cellular physiology. This extends beyond the classical concept of electric remodeling, stressing that alterations of spatial compartmentation of ion channels and receptors are responsible for pathology, in addition to classically appreciated changes in protein expression and posttranslational modifications.

### Limitations

In the present study, we used MβCD to disrupt caveolae structures that might have some potential side effects on cellular electrophysiology. However, it is the only available approach to physically destroy caveolae at the moment and it is widely used by many groups, without significant effects on T-tubule structure.^13,17,26^ Accordingly, in our study, after 30 minutes treatment with 10 mM MβCD, no changes in cell topography and T-tubule density were found (online-only Data Supplement Figure V). In addition, MβCD treatment did not affect T-LTCC biophysical properties: neither T-LTCC occurrence nor their open probability and current-voltage characteristics were altered. Moreover, similar results on LTCC distribution and Ca^2+^ activity were obtained with use of a specific Cav3-targeted LTCC-blocking agent, Rem peptide,^12^ suggesting no direct effect of MβCD on LTCC. Therefore, the influence on the use of MβCD in this study should be limited, if there is any, and will not affect the result and the deducting conclusion regarding the LTCC distribution and functioning.

## Acknowledgments

We thank Peter O’Gara for cardiomyocyte isolation, and Andrei Buzuk for his help with data processing. We are grateful for the generous help provided by Dr Rogers, Electron Microscopy Unit, Royal Brompton Hospital, London, SW3 6NP.

## Sources of Funding

This work was primarily supported by Wellcome Trust WT090594 and British Heart Foundation 12/18/30088 to Dr Gorelik.

## Disclosures

None.

## Supplementary Material

## CLINICAL PERSPECTIVE

Heart failure is one of the most important causes of arrhythmogenic atrial remodeling. The overall risk of atrial arrhythmias increases almost 6-fold when heart failure is present. Despite these troubling statistics, the mechanisms of atrial remodeling in the settings of heart failure are still poorly understood. In this study, we introduced a novel concept of electrophysiological atrial remodeling resulting from changes in the subcellular compartmentation of calcium-signaling complexes, accompanied by a disruption of cellular structure. We provide direct evidence for 2 distinct subpopulations of functional L-type calcium channels in rat and human atrial myocytes: one localized in the T-tubules and another linked to caveolae structures. Such spatial compartmentation is believed to impact calcium channel function and regulation, which is evident from their distinct role in the control of sarcoplasmic reticulum calcium release in atrial versus ventricular myocytes. We demonstrate a heart failure–dependent and microdomain-specific remodeling of biophysical properties of calcium channels, resulting from a disruption of the delicately balanced dynamic interactions between the channels and their cellular microenvironment. Importantly, this may alter autonomic modulation of channels’ functioning dramatically affecting the excitation-contraction processes and underlying pathophysiology of atrial remodeling. Our findings extend beyond the classical concept of electric remodeling in heart failure and add a new dimension to cardiovascular disease, besides well-acknowledged changes in protein expression and their posttranslational modifications. We anticipate that our assay will be a development platform for more sophisticated therapeutic approaches for atrial-remodeling treatments based on the subcellular distribution of their targets.
