# Supplementary material for: Direct Evidence for Microdomain-Specific Localization and Remodeling of Functional L-Type Calcium Channels in Rat and Human Atrial Myocytes
Source: Circulation. 2015 Dec 21;132(25):2372–84. doi: 10.1161/CIRCULATIONAHA.115.018131 (PMC4689179; doi:10.1161/CIRCULATIONAHA.115.018131)
Supplement: Supplementary file 1 [file cir-132-2372-s001.pdf]

SUPPLEMENTAL MATERIAL

Direct evidence for microdomain–specific localization and remodeling of functional L-type calcium channels in rat and human atrial myocytes

Alexey V Glukhov, Marina Balycheva, Jose L. Sanchez-Alonso, Zeki Ilkan, Anita Alvarez-Laviada, Navneet Bhogal, Ivan Diakonov, Sophie Schobesberger, Markus B. Sikkel, Anamika Bhargava, Giuseppe Faggian, Prakash P. Punjabi, Steven R. Houser, Julia Gorelik

Supplemental Methods

Rat cardiomyocyte isolation and plating

Atrial and ventricular cardiomyocyte isolation was done as previously described[1]. Briefly, Sprague-Dawley rats (150–250 g) were anesthetized with 5% isoflurane-95% O_2 and then killed by cervical dislocation. Hearts were fast extracted and placed in Tyrode solution containing in (mmol/L): 140 NaCl, 6 KCl, 1 $MgCl_2$, 1 $CaCl_2$, 10 glucose and 10 HEPES, adjusted to pH 7.4 with 2 mmol/L NaOH. Using aortic cannulation with the Langendorff setting, the hearts were perfused with Tyrode solution for 5 min, then with low Ca^{2+} solution containing in (mmol/L): 120 NaCl, 5.4 KCl, 5 $MgSO_4$, 5 sodium pyruvate, 20 glucose, 20 taurine, 10 HEPES, 5 nitrilotriacetic acid, and 0.04 $CaCl_2$, adjusted to pH 6.96 with 2 mmol/L NaOH for 5 min, and finally for 10 min with enzyme solution containing in (mmol/L): 120 NaCl, 5.4 KCl, 5 $MgSO_4$, 5 sodium pyruvate, 20 glucose, 20 taurine, 10 HEPES, and 0.2 $CaCl_2$, pH 7.4 with collagenase (1 mg/ml; Worthington) and hyaluronidase (0.6 mg/ml; Sigma-Aldrich). Both left and right atria were then removed, cut into small pieces, re-suspended in enzyme solution, but containing only collagenase (1 mg/ml; Worthington) and shaken in a water bath at 37°C for 20 minutes. Then the achieved cells were filtered through a 200-μm nylon mesh and left in buffer solution at room temperature without centrifuge.

Cardiomyocytes were plated on MatTek dishes coated with laminin and left to stick to bottom for at least 45 minutes before experiments. Cardiomyocytes were used on the same day as isolation. Cells were washed twice with the external recording solution and mounted on the microscope stage for recordings.

Heart failure rat model and cardiomyocyte isolation

Adult male Sprague-Dawley rats (250-300g) underwent proximal coronary ligation to induce chronic myocardial infarction as described before[2]. Briefly, rats were anesthetized with 2%

isoflurane, intubated, and ventilated after preoperative buprenorphine (0.03 mg/kg SC) injection. The thorax was shaved and sterilized with 2% w/v chlorhexidine gluconate in 70% v/v isopropyl alcohol. A left thoracotomy was performed, and the left anterior descending coronary artery was ligated with 6-0 silk. Sham ligation was used as control. Sixteen weeks later, in vivo PV analysis was performed using the 2-F Millar microconductance catheter (SPR838; Millar Instruments) via an apical approach under isoflurane (1.5%) anesthesia. Steady-state data (left ventricle end systolic and diastolic dimensions, and left ventricle ejection fraction) were recorded after 15 minutes' stabilization. Data were recorded using CHART 5.5 software (AD Instruments) and analyzed off line using PVAN 3.6 software (Millar Instruments). Hearts were explanted, weighed, and prepared for cell isolation.

This heart failure model recapitulates many features of chronic heart failure in patients including adverse remodeling of the organ, characterized by left ventricle and left atria dilatation, reduced ejection fraction, raised filling pressures and elevated serum natriuretic peptides[2, 3]. In particular, heart failure rats exhibited left ventricle dilatation, reduced ejection fraction and raised filling pressures (**Supplemental Table 1**). In these rats, the high burden of both atrial[4] and ventricular[5] arrhythmias and an incidence of sudden cardiac death[5] have been previously reported. Ventricular cells from this model have been studied extensively and are well characterized at structural, biochemical, molecular, Ca^{2+} handling, and electrophysiological levels[2, 6, 7].

Patients groups

Human myocytes were isolated from the right atrial appendage left-over samples (n=7, average age 71.4±2.2 years, 5 males and 2 female) obtained during coronary artery bypass surgery (n=5) and mitral valve replacement (n=2) procedure at Hammersmith Hospital, Imperial College London, UK. All the patients had a normal left ventricle function (ejection fraction >60%) and no evidence of atrial arrhythmias. The collected samples were cardioplegically arrested and cooled to 4-7°C in the operating room following cross-clamping of the aorta. The samples were maintained at 4-7°C to preserve tissue during 10 minutes delivery from the operating room to the research laboratory.

Human cardiomyocyte isolation

Human cardiomyocytes were isolated by enzymatic digestion as previously described[2]. Briefly, individual specimens were transferred to ice-cold calcium free Krebs-Ringer saline solution consisting of (in g/L): 7.012 NaCl, 0.402 KCl, 1.332 $MgSO_4$, 0.55 Pyruvate, 3.603 Glucose, 2.502 Taurine, 2.383 HEPES, 1.286 Nitrillotriacetic Acid; pH = 6.96. Connective and adipose tissue were removed and approximately 500mg of myocardial tissue was

minced with razor blades in small cubes (approx. 1-2 mm^3). Then, the tissue pieces were washed with fresh Ca^{2+}-free Krebs-Ringer solution 3 times for 3 min each at 37°C. After wash, cardiac tissue was incubated for 25 min in 10ml of Krebs-Ringer solution containing (in g/L): NaCl 7.012, KCl 0.402, $MgSO_4$ 1.332, Pyruvate 0.55, Glucose 3.603, Taurine 2.502, HEPES 2.383; pH = 7.4, supplemented with 200 nM $CaCl_2$ and Proteinase type XXIV (0.36mg/ml; Sigma-Aldrich) under gentle agitation. The partially digested tissue was transferred to 10ml of Krebs-Ringer saline supplemented with collagenase type XIV (1mg/ml Sigma-Aldrich). The tissue was incubated thrice with this solution for 10 min each at 37°C with gentle agitation. Usually, cardiomyocytes were visible by phase contrast light microscopy after the first incubation step, with the biggest amount of cells after the second incubation step. After each incubation step, the supernatants were transferred to a tube and centrifuged at 600 rpm for 3 min. The pellets were re-suspended in 2-3 mL of Krebs-Ringer solution. After isolation, human cardiomyocytes were plated following the same protocol as rat cardiomyocytes.

Whole mount immunofluorescence labeling

In intact atria tissue, we measured T-tubule organization in a manner similar to those previously reported. For this, whole mount preparation of right and left atria was used as described previously[8]. Briefly, the heart was cannulated and retrogradely perfused with Tyrode solution. The ventricles were dissected away, and the atria were stretched and then pinned to the bottom of a Sylgard-coated chamber and superfused with Tyrode solution. The medial limb of the crista terminalis was cut to open the right atrium appendage. Tissue sections were fixed in freshly prepared 4% paraformaldehyde (PFA) for 30 min before incubation with wheat germ agglutinin (WGA), Alexa Fluor 488 conjugate prepared in PBS (Invitrogen, Glasgow, UK) at 20 µg/ml for 2 hours to visualize T tubules. Mounted whole mount preparations were analyzed with an Olympus FV 1000 Spectral Confocal microscope under a 10X objective; Z stacks were collected and integrated for final 3D images.

T-tubule Labeling

T-tubule density was measured after sarcolemmal membrane labeling with Di-8-ANEPPS as described previously[9]. Cardiomyocytes were incubated with 10 mM Di-8-ANEPPS (Molecular Probes, Eugene, OR, USA) for 1 min and then washed for 3 min before being observed under the confocal microscope. After Di-8-ANEPPS labeling, the density of T-tubules was quantified by the ratio of T-tubule fluorescence (T-tubule membrane) to total plasma membrane fluorescence (total membrane) in the same confocal slice, with excitation at 488 nm and emission detected at 520nm[10]. The T-tubule density was calculated by converting the Di-8-ANEPPS signal to a binary signal, using the autothreshold function of

ImageJ. After exclusion of the surface sarcolemma, the whole z-series was analysed to provide the percentage stained. This was represented as T-tubule density.

3D visualization of T-tubules

Reconstructions of the T-tubular network were performed from confocal stack images. To obtain high-quality visualizations, images were processed with ImageJ[11]. After contrast enhancement (2% saturated pixels), the T-tubular network was manually traced on each image. A threshold was applied and a 3D view was generated using the 3D plugin. The graphical models were then created using the freely-available software Art of Illusion (http://www.artofillusion.org/).

Scanning ion conductance microscopy (SICM)

SICM is a non-contact scanning microscopy technique based on the principle that the flow of ions through the nanopipette filled with electrolytes decreases when the pipette reaches the surface of the sample[12-14]. The result is a three dimensional topography image of live cells with resolution of up to ≤ 20nm[15]. All topographical images in this study were recorded using a variant of SICM called hopping probe ion conductance microscopy[16], implemented on a software platform that controls the ICnano sample scan system13 (Ionscope Ltd, UK). The scan head of the ICnano system consists of a three axis piezo-translation system (Physik Instrumente, UK) with a 100µm x 100µm x–y piezo-stage for sample positioning and 38µm z-axis piezo-actuator for the vertical movement of the pipette, mounted on the stage of a conventional inverted microscope (Diaphot 200, Nikon Corporation, Tokyo, Japan). Glass nanopipettes of ~100nm ID pulled from 1.0 mm O.D. 0.5 mm I.D. borosilicate capillary were used in all experiments. Axopatch 200Apatch-clamp amplifier (Molecular Devices, USA) was used to measure the pipette current as well as to record ion channel activity. Cell-attached currents were digitized using Digidata 1200B and acquired using pClamp 10 data acquisition system (Axon Instruments; Molecular devices).

Super-resolution scanning patch-clamp with pipette clipping modification

After generating a topographical image of the cell surface by SICM, the tip diameter of the pipette is widened by clipping[17] to increase the area of attachment. Controlled widening of the scanning nano-pipette tip is described in details in [17]. Briefly, the nanopipette tip-clipping procedure consisted of three steps. First, the pipette was navigated to a previously identified area of the coverslip free of cardiomyocytes. Second, the fall rate (the rate at which the pipette repeatedly approaches the surface during ‘‘hopping’’) was increased from the standby rate (typically 60 nm/ms) by approximately one order of magnitude (to ~500 nm/ms). At this fall rate, the noncontact mode of hopping probe could no longer be preserved

because of the inherent latency of the z axis piezo feedback control. As a result, the pipette repeatedly crashed into the coverslip, clipping its tip and increasing its diameter because of the conical shape of the pipette. Pipette tip clipping resulted in stepwise increases of the pipette current as its resistance dropped[17]. The clipping was automatically stopped by returning the fall rate to baseline (60 nm/ms) once the pipette current reached a desired level. This process could be repeated to fine-tune the desired pipette tip diameter in steps as small as 10% by varying the stop criteria for current increase, duration and ‘‘clipping’’ fall rate.

Importantly the controlled clipping procedure did not change the overall shape of the pipette tip but reliably allowed the inner tip diameter to be increased approximately 4-fold: from 107±16nm to 417±48nm[17]. The experimentally determined relationship between pipette resistance and inner pipette tip diameter for both intact and widened pipettes was in close agreement with theoretical predictions based on the tip geometry. On average the resistance of the widened pipettes was decreased ~2.4-fold (from 92.2 ± 8.9 MU to 38.7 ± 4.0 MU), thus making the modified pipettes more suitable for whole-cell patch-clamp recordings. The pipette is then lowered to a specific location (T-tubule or crest) until it touches the membrane and a gigaseal is established.

Single channel electrophysiological recordings and data analysis

Cell-attached patch-clamp recordings were performed at room temperature as previously described[18]. Single-channel recordings were obtained using super-resolution scanning patch-clamp method with pipette clipping modification. After generation of a topographical image of the cell surface by SICM, the tip diameter of the pipette was widened from ~100 to ~350nm thereby increasing the surface area underneath and thus increasing the probability of successful recordings[17, 18]. The increase in the tip diameter was ascertained by an increase in the pipette resistance, in this case, from 80 MΩ to 30 MΩ. After clipping, the pipette was moved back and placed to a precise coordinate, centered at a site of known topology, T-tubule opening or crest of sarcolemma, and then sealed to the membrane for recording of functional LTCCs in cell-attached mode. Then non-contact mode of SICM was switched off and the pipette was lowered using the piezo actuator until it touched the cell membrane. Negative pressure was applied to get a giga-seal between the pipette and the membrane. All electrophysiological recordings were performed in the cell-attached patch-clamp configuration with resistance more than 4 GΩ and a current leak less than 2 pA. For recording of Ca^{2+} channels, cardiomyocytes were bathed in an external solution containing in (mmol/L): 120 K-gluconate, 25 KCl, 2 $MgCl_2$, 1 $CaCl_2$, 2 EGTA, 10 Glucose, 10 HEPES, pH 7.4 with NaOH, ~300 mOsm. Pipettes (borosilicate glass, 25-30 MOm) were filled with an

internal recording solution containing in (mmol/L): 90 $BaCl_2$, 10 HEPES, 10 Sucrose, pH 7.4 with TEA-OH, ~280 mOsm. Axopatch 200A patch-clamp amplifier (Molecular Devices, USA) were used to record ion channel activity. Cell-attached currents were digitized using Digidata 1200B and acquired using a pClamp 10 data acquisition system (Axon Instruments; Molecular devices).

Single L-type Ca^{2+} channels (LTCCs) were identified and characterized by their voltage dependent properties. For this purpose depolarizing pulses from a holding potential of -80 mV were elicited to test potentials between -20 and + 20 mV. Analysis was performed as previously described[18]. Single channels were sampled at 10 kHz and filtered at 2 kHz (- 3 dB, 8-pole Bessel). Single channel data were analysed using Clampfit version 10.2. Channel conductance was calculated by plotting the amplitude of fully resolved openings against the test potential for every single experiment. Applied voltage was corrected for a liquid junction potential of -16.7 mV.

Open probability was calculated from at least 10-15 consecutive sweeps. Peak current was calculated as the maximum of the overall average current from one single channel. In case of double- or triple-channel patches, *n* was derived from the maximum current amplitude divided by the number of channels in the patch.

Single channel current recordings were initially examined for presence of sub-conductance states using all points' histograms. Most patches exhibited three to four conductance levels which were used for analysis and were named as levels 1 to 4. Each recording was analyzed separately for the conductance levels 1-4 using a threshold appropriate to the substate amplitude of openings. Openings that reached the 90% threshold for a given conductance state and events were also smaller than the next sub-conductance level were included. Single brief events shorter than 1ms were not included in the analysis.

Whole-cell electrophysiological recordings

Macroscopic Ca^{2+} currents were recorded using the whole-cell patch-clamp configuration with the external recording solution of the following composition (in mmol/L): 120 Tetraethylammonium-chloride, 10 CsCl, 10 Glucose, 10 HEPES, 1.5 $MgCl_2$, 1 $CaCl_2$, pH 7.4 with CsOH. An internal pipette solution contained (in mmol/L): 100 Cs-methanesulfonate, 40 CsCl, 10 HEPES 5 EGTA, 2 $MgCl_2$, 5 Mg-ATP, 0.75 $MgCl_2$, pH 7.2 with CsOH. Patch pipettes had mean resistances of 3.5–5 MΩ. Currents were recorded using an Axopatch-1D amplifier connected to a Digidata1322A acquisition system (Axon Instruments, Foster City, CA, USA). The bath was connected to the ground via an Ag–AgCl pellet. Data were low-

pass filtered at 2 kHz using the built-in Bessel filter of the amplifier and sampled at 10 kHz. All recordings were performed at room temperature (22-24°C). $I_{Ca,L}$ channel activity was recorded during 200 ms from a holding potential of -40 mV to test potentials ranging from -40 to +60 mV, with pulses applied every 2s in 5 mV increments. Results were analyzed offline using pCLAMP10 (Axon Instruments) and OriginPro8.6 (OriginLab) software packages. Series resistance and whole cell capacitance were electronically compensated between 70 and 80% for each cell. Current amplitude at 10 mV was taken as a peak current for each cell. This value was divided by cells capacitance and was termed Ca^{2+} current density. Mean current values ± SEM were plotted as current-voltage (I-V) relationship. *I–V*'s were fitted with the modified Boltzmann equation, $I = [G_{max} \times (V_m - E_{rev})]/\{1 + \exp[(V_m - V_{0.5a})/K_a]$, where V_m is the test potential, $V_{0.5a}$ is the half-activation potential, E_{rev} is the extrapolated reversal potential, G_{max} is the maximum slope conductance and K_a reflects the slope of the activation curve.

Optical mapping and data analysis

The Ca^{2+}-sensitive fluorescent dye Fluo-4 was used to monitor localized changes in cytoplasmic [Ca^{2+}]. Optical mapping of Ca^{2+} transients was performed with modifications as previously described[2]. Briefly, aliquots of cells were incubated with Fluo-4 AM (10 µmol/L) for 25-30 min. The cells were then superfused with Hanks balanced salt solution for 10 minutes to allow intracellular de-esterification. Fluo-4 was excited using the 488-nm line of an argon laser and the emitted fluorescence collected through a 520-nm filter. Imaging was conducted on an inverted Nikon microscope (Eclipse Ti) equipped with a MiCAM Ultima-L CMOS (SciMedia, USA Ltd., CA) camera and sampled at 1,000-500 frames/sec. The fluorescent signals were amplified, digitized, and visualized during the experiment using specialized software (SciMedia, USA Ltd., CA). A custom-made Matlab-based computer program was used to analyze APs offline[19]. The signals were filtered using the low-pass Butterworth filter at 64 Hz. Ca^{2+} transient activation maps were constructed from activation times which were determined from the $(dF/dt)_{max}$ of each channel, where F is the Ca^{2+} fluorescent signal. Ca^{2+} transient duration was measured as the time from the upstroke to 80% recovery.

To investigate spontaneous Ca^{2+} transient events, pacing frequency was progressively increased from 0.5 Hz up to 4 Hz. Cells were electrically paced at 4 Hz for 1 min to enhance sarcoplasmic reticulum Ca^{2+} loading. Non-propagating Ca^{2+} sparks and propagation throughout the entire cell Ca^{2+} waves were quantified during 8-16 sec rest period after cessation of pacing[20, 21].

Electron microscopy

To visualize surface structures, electron microscopy was applied as previously described[22]. Briefly, isolated cardiomyocytes were fixed with 2.5% glutaraldehyde for 2 to 4 h and then centrifuged at 500 *g* for 5 min and the pellet was left overnight. Then a drop of melted 2% agarose was carefully added to the pellet without disturbing and let to gel, following by fixing with gluteraldehyde for at least overnight. The pellet was washed three times in cacodylate buffer and fixed in 1% osmium-tetroxide, followed by a 5-10 min washing with pure water. The samples were dehydrated through a series of graded alcohols, propylene oxide, and embedded in araldite. For low power examination by light microscopy before EM examination, 1 µm thick sections were cut and stained with 1% toluidine blue in 1% borax. For transmission electron microscopy, ultra-thin sections were stained with uranyl acetate and lead citrate. The ultrastructural features of cardiac myocytes, especially the membrane area were examined.

Cell culture and adenoviral transduction

Adenovirus constructs co-expressing GFP and $Rem^{1\text{-}265}$ were used. In addition, the cytosolic truncation mutant $Rem^{1\text{-}265}$ was used[23, 24]. Deletion of the conserved, polybasic C-terminus after residue 265 prevented efficient localization of Rem to the plasma membrane and eliminated inhibition of $Ca_V1.2$ activity[24, 25]. To specifically target Rem to caveolae, a canonical caveolin binding domain, RNVPPIFNDVYWIAF 4 was fused to $Rem^{1\text{-}265}$ and $Rem^{1\text{-}265}$-Cav was created[26]. As shown previously in rat ventricular myocytes, $Rem^{1\text{-}265}$-Cav localized to plasma membrane specifically within caveolin-containing lipid rafts, rather than lipid rafts in general, and did not displace molecules normally found in caveolae[26].

Isolated atrial myocytes were washed (3X) with a serum-free medium (Medium 199, Sigma) supplemented with penicillin, streptomycin and gentamycin and plated on laminin-coated glass cover-slips or culture plates[26]. Myocytes were then infected with adenovirus expressing $Rem^{1\text{-}265}$/GFP and $Rem^{1\text{-}265}$-Cav for 12 hours at a multiplicity of infection of 100. During the experimental period, culture media was changed once per day. Infection efficiency was determined 48 hours after infection by GFP fluorescence intensity and was typically assessed to be 60-65%.

REFERENCES

1. Vescovo G, Jones SM, Harding SE, Poole-Wilson PA. Isoproterenol sensitivity of isolated cardiac myocytes from rats with monocrotaline-induced right-sided hypertrophy and heart failure. *J Mol Cell Cardiol*. 1989;21:1047-1061.

2. Lyon AR, MacLeod KT, Zhang Y, Garcia E, Kanda GK, Lab MJ, Korchev YE, Harding SE, Gorelik J. Loss of t-tubules and other changes to surface topography in ventricular myocytes from failing human and rat heart. *Proc Natl Acad Sci USA*. 2009;106:6854-6859.

3. Yanni J, Tellez JO, Maczewski M, Mackiewicz U, Beresewicz A, Billeter R, Dobrzynski H, Boyett MR. Changes in ion channel gene expression underlying heart failure-induced sinoatrial node dysfunction. *Circulation Heart Fail*. 2011;4:496-508.

4. Cardin S, Guasch E, Luo X, Naud P, Le Quang K, Shi Y, Tardif JC, Comtois P, Nattel S. Role for microrna-21 in atrial profibrillatory fibrotic remodeling associated with experimental postinfarction heart failure. *Circulation Arrhythm Electrophysiol*. 2012;5:1027-1035.

5. Lyon AR, Bannister ML, Collins T, Pearce E, Sepehripour AH, Dubb SS, Garcia E, O'Gara P, Liang L, Kohlbrenner E, Hajjar RJ, Peters NS, Poole-Wilson PA, Macleod KT, Harding SE. SERCA2a gene transfer decreases sarcoplasmic reticulum calcium leak and reduces ventricular arrhythmias in a model of chronic heart failure. *Circulation Arrhythm Electrophysiol*. 2011;4:362-372.

6. Lyon AR, Nikolaev VO, Miragoli M, Sikkel MB, Paur H, Benard L, Hulot JS, Kohlbrenner E, Hajjar RJ, Peters NS, Korchev YE, Macleod KT, Harding SE, Gorelik J. Plasticity of surface structures and beta(2)-adrenergic receptor localization in failing ventricular cardiomyocytes during recovery from heart failure. *Circulation Heart Fail*. 2012;5:357-365.

7. Del Rio R, Marcus NJ, Schultz HD. Carotid chemoreceptor ablation improves survival in heart failure: Rescuing autonomic control of cardiorespiratory function. *J Am Coll Cardiol*. 2013;62:2422-2430.

8. Glukhov AV, Kalyanasundaram A, Lou Q, Hage LT, Hansen BJ, Belevych AE, Mohler PJ, Knollmann BC, Periasamy M, Gyorke S, Fedorov VV. Calsequestrin 2 deletion causes sinoatrial node dysfunction and atrial arrhythmias associated with altered sarcoplasmic reticulum calcium cycling and degenerative fibrosis within the mouse atrial pacemaker complex1. *Eur Heart J*. 2015;36:686-697.

9. Kawai M, Hussain M, Orchard CH. Excitation-contraction coupling in rat ventricular myocytes after formamide-induced detubulation. *Am J Physiol*. 1999;277:H603-609.

10. Louch WE, Bito V, Heinzel FR, Macianskiene R, Vanhaecke J, Flameng W, Mubagwa K, Sipido KR. Reduced synchrony of Ca^{2+} release with loss of t-tubules-a comparison to Ca^{2+} release in human failing cardiomyocytes. *Cardiovasc Res*. 2004;62:63-73.

11. Schindelin J, Arganda-Carreras I, Frise E, Kaynig V, Longair M, Pietzsch T, Preibisch S, Rueden C, Saalfeld S, Schmid B, Tinevez JY, White DJ, Hartenstein V, Eliceiri K, Tomancak P, Cardona A. Fiji: An open-source platform for biological-image analysis. *Nat Methods*. 2012;9:676-682.

12. Korchev YE, Milovanovic M, Bashford CL, Bennett DC, Sviderskaya EV, Vodyanoy I, Lab MJ. Specialized scanning ion-conductance microscope for imaging of living cells. *J Microscopy*. 1997;188:17-23.

13. Korchev YE, Bashford CL, Milovanovic M, Vodyanoy I, Lab MJ. Scanning ion conductance microscopy of living cells. *Biophys J*. 1997;73:653-658.

14. Hansma PK, Drake B, Marti O, Gould SA, Prater CB. The scanning ion-conductance microscope. *Science*. 1989;243:641-643.

15. Shevchuk AI, Frolenkov GI, Sanchez D, James PS, Freedman N, Lab MJ, Jones R, Klenerman D, Korchev YE. Imaging proteins in membranes of living cells by high-resolution scanning ion conductance microscopy. *Angewandte Chemie*. 2006;45:2212-2216.

16. Novak P, Li C, Shevchuk AI, Stepanyan R, Caldwell M, Hughes S, Smart TG, Gorelik J, Ostanin VP, Lab MJ, Moss GW, Frolenkov GI, Klenerman D, Korchev YE. Nanoscale live-cell imaging using hopping probe ion conductance microscopy. *Nat Methods*. 2009;6:279-281.

17. Novak P, Gorelik J, Vivekananda U, Shevchuk AI, Ermolyuk YS, Bailey RJ, Bushby AJ, Moss GW, Rusakov DA, Klenerman D, Kullmann DM, Volynski KE, Korchev YE. Nanoscale-targeted patch-clamp recordings of functional presynaptic ion channels. *Neuron*. 2013;79:1067-1077.

18. Bhargava A, Lin X, Novak P, Mehta K, Korchev Y, Delmar M, Gorelik J. Super-resolution scanning patch clamp reveals clustering of functional ion channels in adult ventricular myocyte. *Circ Res*. 2013;112:1112-1120.

19. Lou Q, Fedorov VV, Glukhov AV, Moazami N, Fast VG, Efimov IR. Transmural heterogeneity and remodeling of ventricular excitation-contraction coupling in human heart failure. *Circulation*. 2011;123:1881-1890.

20. Hohendanner F, Walther S, Maxwell JT, Kettlewell S, Awad S, Smith GL, Lonchyna VA, Blatter LA. Inositol-1,4,5-trisphosphate induced Ca^{2+} release and excitation-contraction coupling in atrial myocytes from normal and failing hearts. *J Physiol*. 2014.

21. Voigt N, Li N, Wang Q, Wang W, Trafford AW, Abu-Taha I, Sun Q, Wieland T, Ravens U, Nattel S, Wehrens XH, Dobrev D. Enhanced sarcoplasmic reticulum Ca^{2+} leak and increased Na^{+}-Ca^{2+} exchanger function underlie delayed afterdepolarizations in patients with chronic atrial fibrillation. *Circulation*. 2012;125:2059-2070.

22. Wright PT, Nikolaev VO, O'Hara T, Diakonov I, Bhargava A, Tokar S, Schobesberger S, Shevchuk AI, Sikkel MB, Wilkinson R, Trayanova NA, Lyon AR, Harding SE, Gorelik J. Caveolin-3 regulates compartmentation of cardiomyocyte beta2-adrenergic receptor-mediated cAMP signaling. *J Mol Cell Cardiol*. 2014;67:38-48.

23. Finlin BS, Crump SM, Satin J, Andres DA. Regulation of voltage-gated calcium channel activity by the Rem and rad GTPases. *Proc Natl Acad Sci USA*. 2003;100:14469-14474.

24. Correll RN, Pang C, Finlin BS, Dailey AM, Satin J, Andres DA. Plasma membrane targeting is essential for Rem-mediated Ca^{2+} channel inhibition. *J Biol Chem*. 2007;282:28431-28440.

25. Heo WD, Inoue T, Park WS, Kim ML, Park BO, Wandless TJ, Meyer T. PI(3,4,5)P3 and PIi(4,5)P2 lipids target proteins with polybasic clusters to the plasma membrane. *Science*. 2006;314:1458-1461.

26. Makarewich CA, Correll RN, Gao H, Zhang H, Yang B, Berretta RM, Rizzo V, Molkentin JD, Houser SR. A caveolae-targeted L-type Ca^{2+} channel antagonist inhibits hypertrophic signaling without reducing cardiac contractility. *Circ Res*. 2012;110:669-674.

Supplemental Table 1. Functional characterisation of sham-operated age-matched control (AMC) and heart failure (HF) rats

Characteristic	AMC (n=6)	HF (n=14)	P-value
Body weight, g	592± 33	609 ± 54	0.486
Heart weight, g	1.6 ± 0.2	2.0 ± 0.3	0.004
Heart weight/body weight, mg/g	2.7 ± 0.2	3.3 ± 0.4	0.005
LV end dyastolic dimension, mm	8.2 ± 0.7	10.3 ± 0.6	<0.001
LV end systolic dimension, mm	4.1 ± 0.7	8.4 ± 0.8	<0.001
LV ejection fraction, %	79.7 ± 5.1	36.3 ± 8.8	<0.001
LA diameter, mm	5.3 ± 1.0	7.0 ± 0.5	<0.01

LV indicates left ventricle; LA, left atrium.

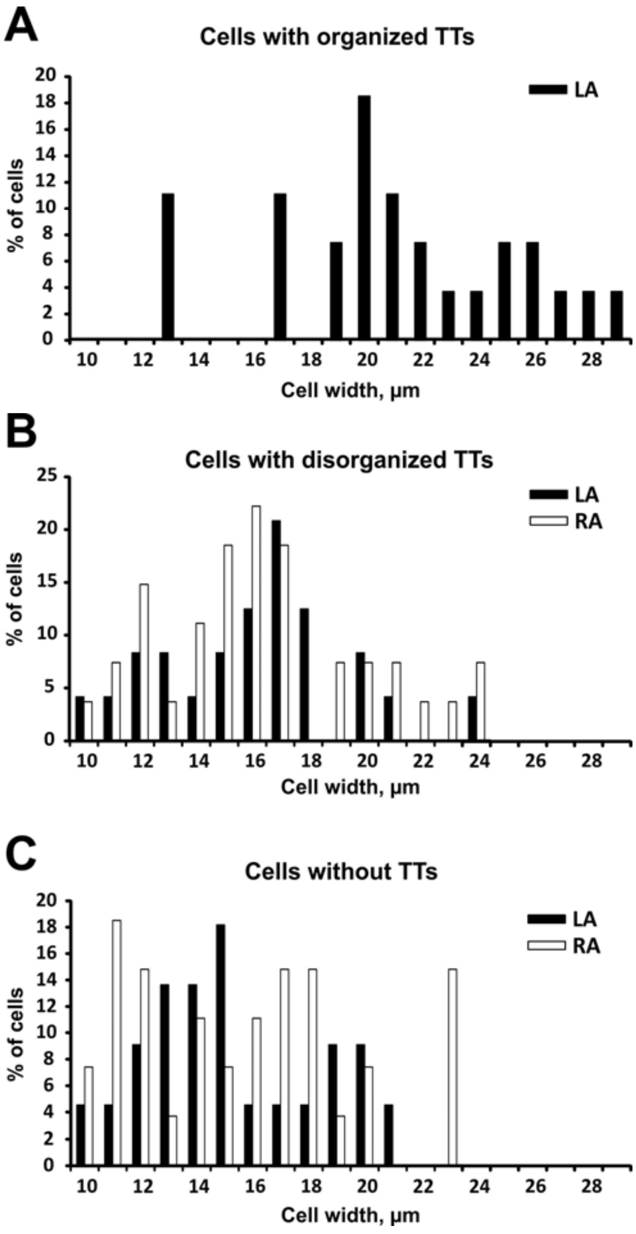

Supplemental Figure 1. Cells with T-tubules are larger and the left atrium has more cells with T-tubules. Histograms of cell widths measured for left and right atria for three groups of atrial cardiomyocytes with: organized T-tubules (A), disorganized T-tubules (B), and absent T-tubules (C).

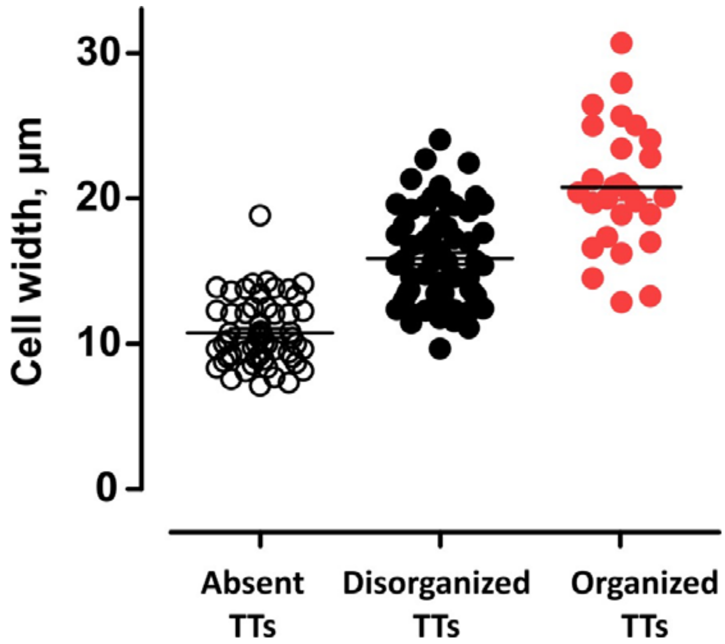

Supplemental Figure 2. Cell width measured in atrial myocytes with different T-tubule structure. Cell widths for individual myocytes with organised T-tubules (TTs, red circles), disorganized TTs (black circles), and absent TTs (empty circles) are shown. Average values are shown by horizontal lines for each group (average cell width: 13.0 ± 0.4 µm, 15.9 ±0 .4 µm, and 20.7 ± 0.8 µm for cells with absent TTs, disorganized TTs, and organized TTs, respectively). P<0.001 by unpaired Student t-test between all groups.

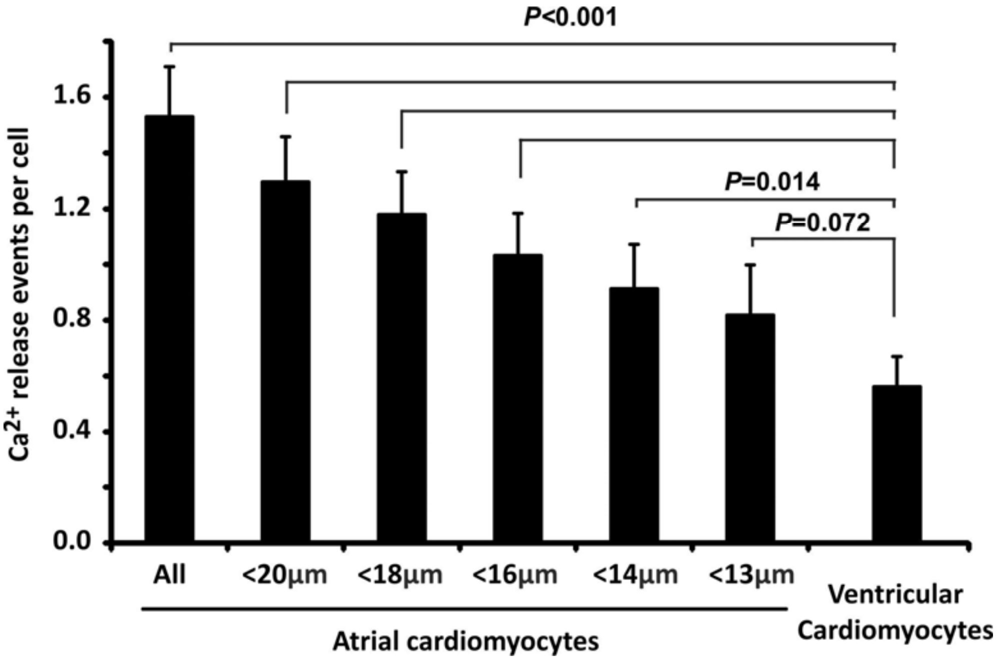

Supplemental Figure 3. Dependence of spontaneous Ca^{2+} release events from cell width. Number of spontaneous Ca^{2+} release events per cell was measured for all atrial cardiomyocytes and for atrial cardiomyocytes of different cell width. Note that number of Ca^{2+} release events significantly decreases with cell width narrowing. For atrial cardiomyocytes thinner than 13 µm, number of Ca^{2+} release events did not differ from that measured in ventricular cardiomyocytes.

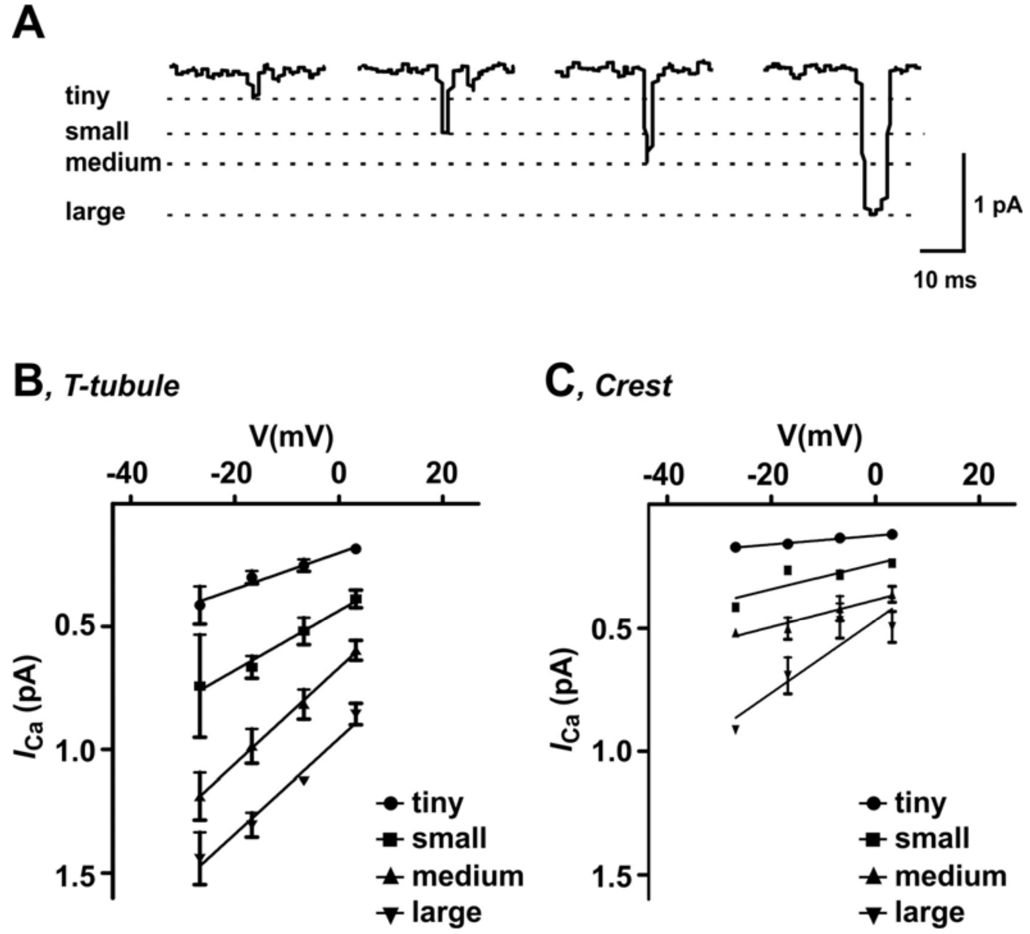

Supplemental Figure 4. Single channel current-voltage (*I*-V) relationships for different conductance levels of LTCCs. (A) Representative openings at two voltages for each conductance state, with Ba^{2+} as the charge carrier. Clear openings to three-four distinct conductance levels were observed at -26.7 mV. The LTCCs substates appear to be true openings to smaller conductance states and did not arise from filtering artefacts. Openings to the substates were well resolved, greatly exceeding the filter rise time (average open time of the small substate = 2.98 ± 0.20 ms (n=127), shorted open time = 1.1 ms; filter rise time = 100 μs), so they were not produced by unresolved transitions of relatively slow interconversions between open and closed conformations. Indeed, the amplitude of all conduction states was voltage-dependent (B-C).

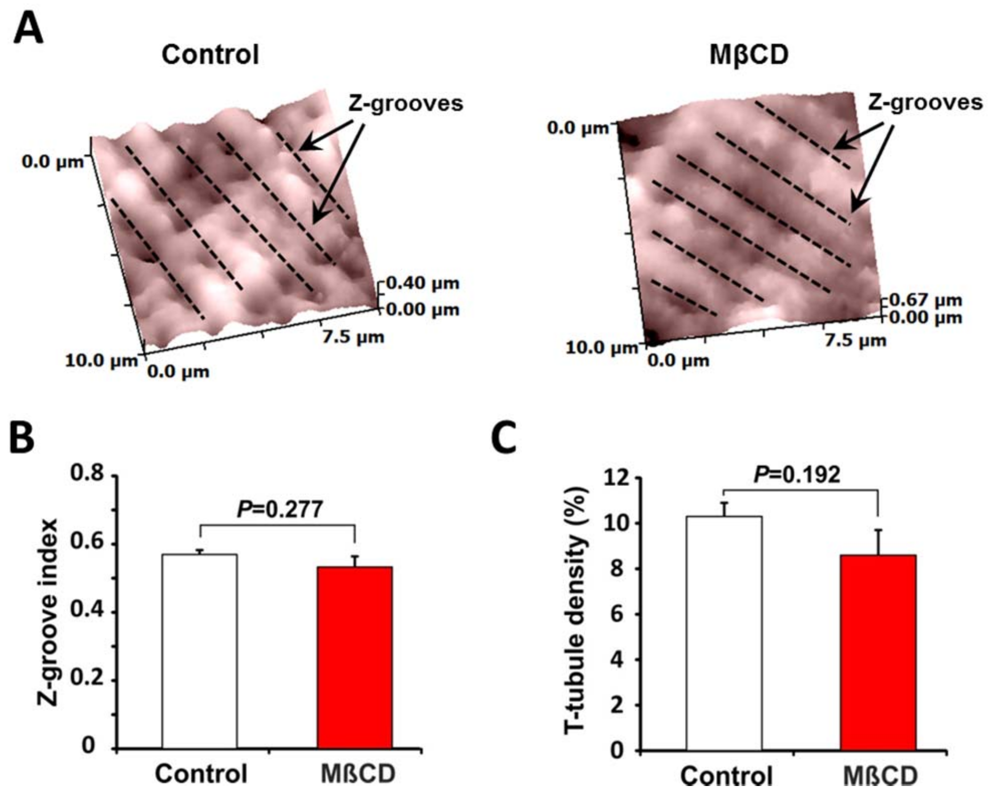

Supplemental Figure 5. Ultrastructural changes in rat atrial myocytes after methyl-β-cyclodextrin incubation. (A) Representative SICM scans of untreated (*left*) and MßCD treated (*right*) rat atrial myocytes. Z-grooves are shown by dotted lines on the scans. (B) Average Z-groove index measured for rat atrial myocytes before (n=82 cells) and after (n=26 cells) MßCD treatment. (C) T-tubule system density measured in untreated (n=112) and MßCD treated (n=34) rat atrial myocytes. Average cell diameter was 14.8 ± 1.4 μm and 14.3 ± 0.6 μm in MβCD group vs 15.6 ± 0.6 μm and 14.9 ± 0.7 μm in control group for cells with disorganized and absent T-tubules, respectively, *NS*.

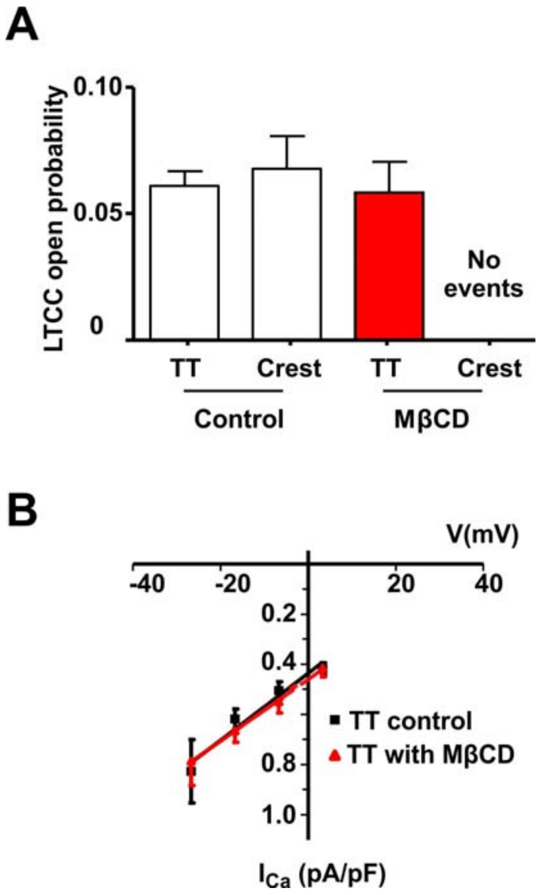

Supplemental Figure 6. Effect of cholesterol depletion through 30 min incubation with 10mM methyl-β-cyclodextrin (MßCD) on LTCC open probability (A) and current-voltage relationship (B).

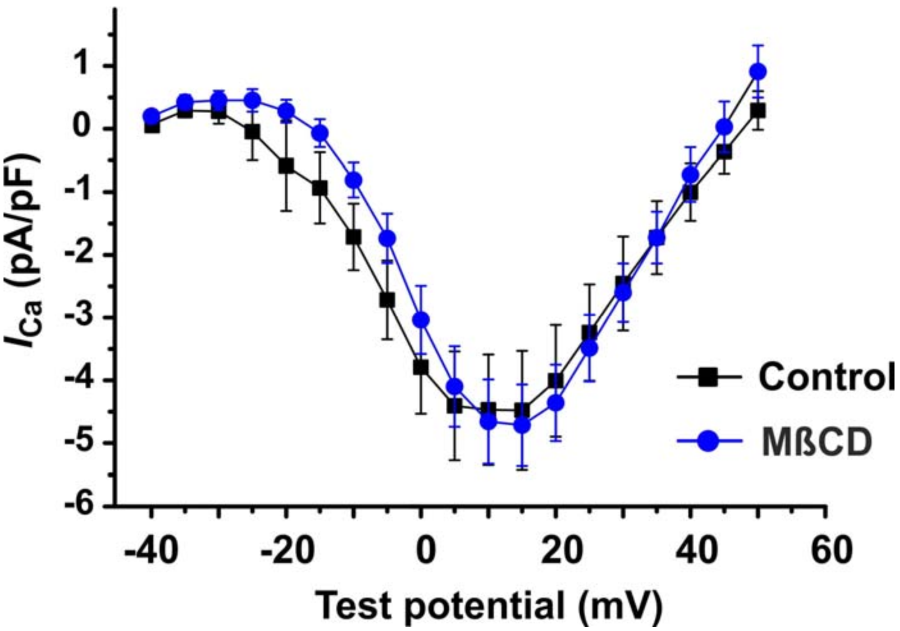

Supplemental Figure 7. Effect of methyl-β-cyclodextrin (MßCD) treatment on ventricular myocytes whole cell current-voltage relationship. N=10 for untreated (control) ventricular myocytes; n=8 for 10 mM MßCD treated ventricular myocytes.

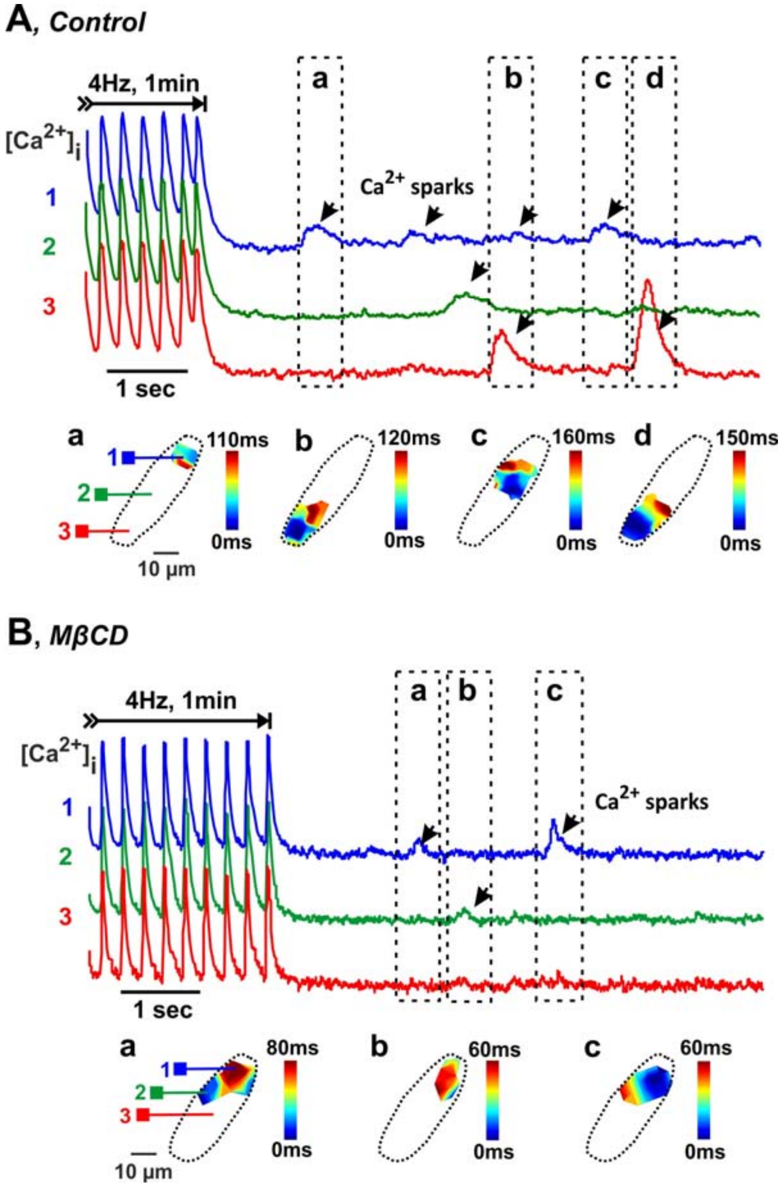

Supplemental Figure 8. Suppression of spontaneous Ca^{2+} sparks after caveolae disruption by cholesterol depletion. Representative optical mapping recordings of Ca^{2+} transients during spontaneous Ca^{2+} sparks evoked by Ca^{2+} overload induced by pacing at 4 Hz for 1 minute. Optical recordings were obtained before (A) and after 30min pre-treatment with methyl-β-cyclodextrin, MßCD (B). Top, optical traces are shown from three different areas (1-3) from the selected cardiomyocytes. Below the traces, Ca^{2+} transient propagation color contour maps are presented for spontaneous Ca^{2+} sparks recorded from the atrial myocyte before and after MßCD treatment.

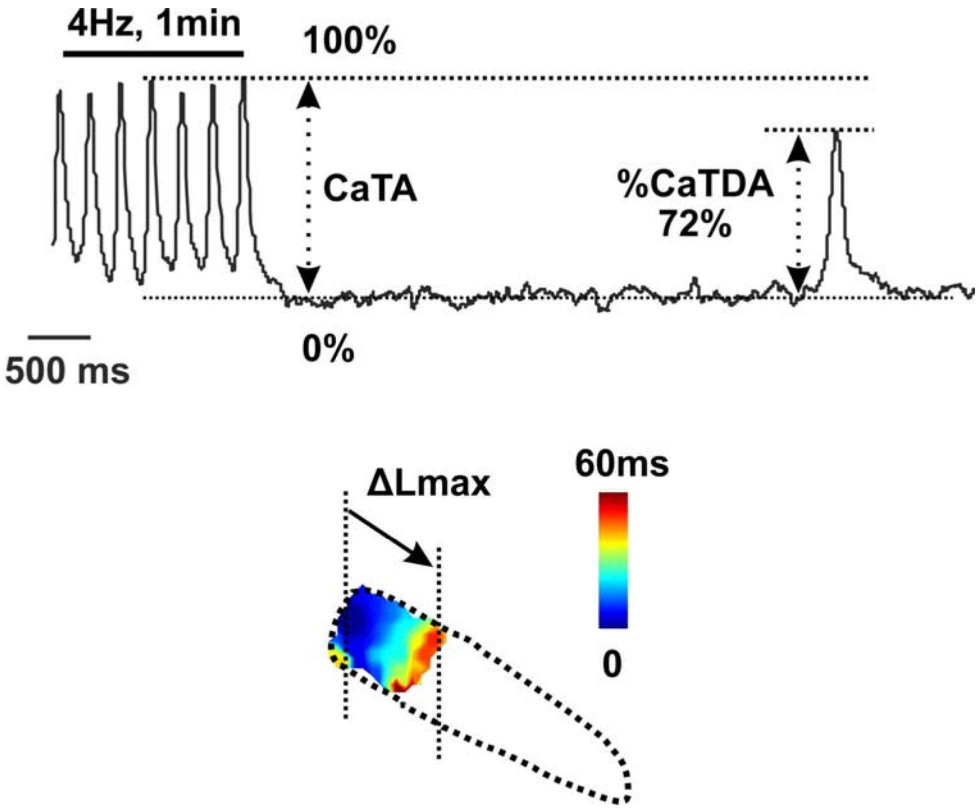

Supplemental Figure 9. Spatio-temporal characteristics of spontaneous Ca^{2+} events recorded in atrial myocytes. Maximal amplitude of events (in % from paced Ca^{2+} transient amplitude, CaTA) and maximal distance activated by the Ca^{2+} release event (ΔLmax, pixels) through the cell were calculated.

Legends for the Video files.

Supplemental Movie 1. 3D reconstruction of sub-cellular T-tubules from atrial cardiomyocyte with organized T-tubule network. T-tubules were visualized by surface membrane stating with with lipophilic membrane indicator Di-8-ANEPPS. A 4 μm x 10 μm x 10 μm area from confocal stack images was used.

Supplemental Movie 2. 3D reconstruction of sub-cellular T-tubules from atrial cardiomyocyte with disorganized T-tubule network. T-tubules were visualized by surface membrane stating with with lipophilic membrane indicator Di-8-ANEPPS. A 15 μm x 10 μm x 2 μm area from confocal stack images was used, as sections of this size best depict the specificities of the T-tubule network in this cell.
